# Nursing Interventions to Promote Health Literacy in Children and Adolescents: A Scoping Review

**DOI:** 10.3390/healthcare14131829

**Published:** 2026-06-24

**Authors:** Catarina Fragoso, Marina Sousa, Fernanda Loureiro, Zaida Charepe

**Affiliations:** 1 Unidade de Cuidados de Saúde Personalizados de Odivelas, Av. Prof. Dr. Augusto Abreu Lopes 36, 2675-297 Odivelas, Portugal; catarina_fragoso_94@hotmail.com; 2 Clínica CUF Almada, Rua Manuel Tito de Morais 2, Monte de Caparica, 2825-146 Caparica, Portugal; marinasousa@rocketmail.com; 3 Centro de Investigação Interdisciplinar em Saúde (CIIS), Faculdade de Ciências da Saúde e Enfermagem (FCSE), Universidade Católica Portuguesa (UCP), 1649-023 Lisbon, Portugal; zaidacharepe@ucp.pt

**Keywords:** scoping review, child, adolescent, nursing, health literacy

## Abstract

**Highlights:**

**What are the main findings?**

- Nursing interventions that promote health literacy vary by age. For example, storytelling is used in early childhood, playful educational strategies are used with children, while group and digital interventions are used with adolescents.
- Most interventions occur in schools and focus on health promotion, disease prevention, and self-management.

**What are the implications of the main findings?**

- Nurses play a key role in promoting health literacy throughout healthcare and community settings, from early childhood onwards.
- Standardized assessment tools and long-term studies are needed to assess the impact of interventions.

**Abstract:**

**Background/Objectives**: Health literacy (HL) is recognized as an important social determinant of health. It supports healthy behaviors and effective health management throughout one’s life. For children and adolescents, developing HL influences their well-being, development, and ability to make informed health decisions. Nurses are strategically positioned to promote HL from an early age. To our knowledge, no prior synthesis has specifically examined nurse-led HL interventions targeting pediatric populations, highlighting the originality and relevance of this scoping review. The purpose of this review was to map and characterize nursing interventions aimed at improving HL outcomes in children and adolescents. **Methods**: A scoping review was conducted according to the Joanna Briggs Institute methodology, using a three-step search strategy, and reported in accordance with the PRISMA-ScR guidelines. Searches were conducted in MEDLINE, CINAHL, Scopus, Web of Science, and ProQuest with no date restriction, including studies published in Portuguese, English, or Spanish. Studies involving children and adolescents (ages 0–18) in any healthcare or community setting were eligible. Data on intervention characteristics and HL outcomes were extracted and analyzed descriptively, and no critical appraisal of the included sources was conducted. **Results**: A total of 44 studies were included. Interventions were predominantly school-based and focused on adolescents (n = 26), with a clear gap in early childhood (n = 2). Studies of early childhood primarily used storytelling and reading activities, whereas interventions targeting older children and adolescents more often employed participatory educational strategies, group-based approaches and digital platforms. The most frequently addressed topics were chronic disease management (n = 12), mental health (n = 7), and nutrition (n = 5). HL domains mainly focused on healthcare and health promotion, with fewer studies addressing disease prevention. Most interventions were conducted in school settings (n = 24), highlighting this context over those in primary care, community, and hospital settings. **Conclusions**: The results revealed nursing interventions used to promote HL, particularly in the management of chronic diseases, mental health and nutrition. However, the existing body of research is still limited. Key gaps include the absence of standardized measurement tools and the scarcity of longitudinal studies evaluating long-term outcomes. These limitations constrain the comparability and generalizability of findings, highlighting the necessity of more rigorous, methodologically robust research to support evidence-based practices. This scoping review comprehensively maps nurse-led interventions that promote HL among children and adolescents, identifying key priorities to guide future research in this area.

## 1. Introduction

Health literacy (HL) has emerged as a critical determinant of population health and a fundamental element of health promotion and disease prevention strategies. Unlike health education, HL refers to the skills that enable individuals to access, understand, evaluate, and apply health information. Health education is a structured learning process aimed that aims to improve these skills. Thus, health education is a key strategy for developing HL [1].

HL was originally defined as the ability to read and understand health-related information, but it has since expanded to include the skills needed to navigate complex health systems, evaluate information, and make informed decisions throughout one’s life [2,3]. HL is now considered a multidimensional concept influenced by individual abilities as well as broader social, environmental, and systemic factors [4]. The World Health Organization (WHO) defines HL as “the cognitive and social skills and the ability to access, understand, and use information to promote and maintain good health” [5]. Nutbeam identifies three levels of HL: (1) Functional/Basic Literacy, comprising basic reading and writing skills; (2) Interactive/Communicative Literacy, encompassing advanced cognitive and social skills that enable active participation and adaptation; and (3) Critical Literacy, referring to the ability to critically analyze information and exercise greater autonomy in decision-making [2]. These levels empower individuals to adopt healthy lifestyles, use health services appropriately, and influence health determinants at social, environmental, and economic levels. HL levels (functional, interactive, critical) refer to individual competences [6], while domains (health care, health promotion, and disease prevention) represent the thematic intervention areas [7].

Several literature reviews have examined HL, specifically with regard to children. In 2009, Sanders and colleagues [8] reviewed the literature on the relationship between parent literacy, child literacy and child health outcomes, as well as interventions designed to improve these outcomes in contexts of low literacy. The authors concluded that child and parental literacy appear to be associated with significant health outcomes. Subsequently, Ormshaw et al. [9] sought to compile, analyze, and describe the methodologies and measurement approaches used in childhood and adolescent HL research. They concluded that, despite a growing body of evidence, clear definitions and standardized measurement tools are essential to advancing the field. Building on this, Bröder et al. [10] conducted a systematic review in 2017 to synthesize the current understanding of HL in childhood and youth. They identified HL as a widely recognized complex and multidimensional construct that emphasizes individual competencies while acknowledging the influence of social and contextual determinants. Nevertheless, the authors noted that most conceptual models prioritize cognitive development and largely overlook the specific needs and social contexts of younger children. Few frameworks address those under ten years of age or in primary school settings.

Despite growing academic interest, childhood HL remains a fragmented field with significant knowledge gaps. There is no consensus on the competencies that children and adolescents need to make informed health decisions [10]. Existing models and measurement tools vary considerably, and most are adapted from adult frameworks focusing primarily on functional literacy [11]. Additionally, research has disproportionately focused on adolescents and parental literacy, with younger children being underrepresented [8,9]. Furthermore, there is limited evidence regarding the mapping of nursing interventions to promote HL across pediatric age groups, primarily due to the heterogeneity and predominantly cross-sectional nature of the available studies [12].

Because they frequently interact with pediatric populations in schools, primary care settings, hospitals, and communities, nurses are well positioned to promote HL in children and adolescents. They provide health education, communication support, disease self-management education, preventive counseling, and adapt information to children’s developmental stage and family context. For the purposes of this scoping review, nursing interventions were defined as those in which nurses played a central or leading role in the design, implementation or execution (including nursing students under supervision), even when implemented within multidisciplinary teams. This reflects the collaborative nature of pediatric healthcare practice.

Although HL in children and adolescents has been widely studied, there is a lack of synthesis research synthesizing nursing-led interventions, which play a critical role in all care settings. Previous reviews have primarily focused on conceptualization and measurement, paying little attention to intervention-based evidence, particularly from a nursing perspective.

The purpose of this scoping review is to map and characterize nursing interventions aimed at improving HL outcomes in children and adolescents, addressing the current lack of synthesis focused specifically on nursing-led strategies. Specifically, we want to identify the implementation contexts, characteristics, and reported outcomes. Due to the diversity of populations, interventions, contexts, and study designs, as well as the field’s evolving nature, a scoping review was deemed the most suitable method for systematically mapping existing evidence, identifying key concepts, and highlighting literature gaps.

In this scoping review, HL is approached as a multidimensional concept, structured into three main domains according to the model of Sørensen et al. [3]: health care, health promotion and disease prevention. This categorization enables a clear and comparable analysis of nursing interventions, avoiding ambiguities between levels of competence and thematic areas.

The findings of this scoping review could serve as the basis for the future development of practical guidelines for healthcare professionals on how to promote HL in this population. The results are also expected to inform the education and training of these professionals, ultimately supporting the integration of HL promotion strategies into pediatric clinical practice.

Before conducting this scoping review, we consulted public registration platforms to verify whether any literature reviews on the topic had been registered as ongoing or completed. The searched platforms included the Open Science Framework (OSF), PROSPERO, Figshare, INPLASY, the Cochrane Library of Systematic Reviews, and the Joanna Briggs Institute (JBI) Systematic Review Register. No reviews focusing on nursing interventions to promote HL in children and adolescents were identified.

## 2. Materials and Methods

The scoping review was conducted following the Joana Briggs Institute (JBI) methodology, using a three-step research strategy [13,14,15]: (i) First stage—Preliminary research. An initial search was carried out in the MEDLINE (via PubMed) and CINAHL (via EBSCO) databases to identify relevant articles on nursing interventions for HL in children and adolescents. The terms and keywords from the titles, abstracts, and indexing of the selected articles were analyzed to improve the search strategy; (ii) Second stage—Comprehensive search. Based on the results of the first stage, a detailed search strategy was developed and adapted to each database. Articles published in Portuguese, English, or Spanish with no date restriction were included; Third step—Additional search and selection of sources. Public record platforms (Open Science Framework, PROSPERO, Figshare, INPLASY, Cochrane Library, JBI Systematic Review Register) were searched to identify additional studies.

We used the Population, Concept and Context (PCC) approach as recommended by the JBI [16] to formulate our research question: which nursing interventions promote HL in children and adolescents? (Population: children and adolescents; concept: nursing interventions that promote HL; context: all nursing practice settings, in which direct HL promotion interventions with children and adolescents could occur).

### 2.1. Protocol and Registration

The scoping review protocol was developed following the Preferred Reporting Items for Scoping Reviews (PRISMA-ScR) [17] guidelines and was registered with the OSF (https://osf.io/zv53s/, accessed on 25 August 2025).

To demonstrate compliance with these guidelines, a Supplementary File (Supplementary File S1) is included.

### 2.2. Eligibility Criteria

Population. This scoping review considered articles that included children and adolescents as participants. We used the WHO’s broad definition of children as any human being under the age of 18 [18]. Studies with mixed samples of adolescents and young adults were only considered eligible when the target population explicitly included adolescents, the intervention was directly relevant to adolescent HL, and the mean age of participants did not exceed 18 years. Studies whose target population was exclusively adults, or whose intervention focused only on parents, caregivers, or health professionals, were excluded, even if indirectly related to children’s HL. While we recognize these individuals’ contributions, our aim was to address the perspective of children and adolescents.

Context. We considered primary and hospital healthcare settings, as well as other settings, such as schools and communities.

Concept. The central concept of this review was HL interventions delivered directly to the pediatric population by nurses. To ensure consistency during the selection process, only those interventions that were explicitly described in the publication as having nurses in a central and identifiable role were considered eligible. Studies in which nurses were mentioned solely as members of a multidisciplinary team without clearly specified contributions were excluded. This approach aimed to ensure that the included interventions reflected substantive nursing contributions while acknowledging the collaborative nature of pediatric healthcare.

Classification of nursing interventions in multidisciplinary contexts. To address the frequent inclusion of multiprofessional teams, additional criteria were applied to distinguish nurse-led interventions from broader, multidisciplinary approaches. An intervention was classified as nurse-led if nurses played a central or leading role in designing, delivering, implementing, coordinating, or supervising the intervention. This included interventions delivered by nursing students under the supervision of a registered nurse.

Only multidisciplinary studies that explicitly detailed a distinct and substantive nursing contribution to the intervention were included. In these cases, the scope and nature of nursing practice were clarified using the Nursing Interventions Classification (NIC) for nursing-specific activities. Studies that referred to nurses only as part of the team without specifying their role or contribution were excluded to maintain the focus on nursing-led practice.

Eligible studies were those that described, evaluated, or implemented interventions delivered directly by nurses to children and adolescents, with the aim of improving their ability to access, understand, evaluate, and use health information. Interventions that focused exclusively on training parents, caregivers, or other adults without direct nurse-child interaction were excluded, even when addressing HL.

Study designs. In line with the exploratory nature of this scoping review, we considered a wide variety of study designs, including qualitative, quantitative and mixed methods approaches. Both primary and secondary studies were eligible for inclusion. Specifically, we included experimental and quasi-experimental designs, such as randomized and non-randomized controlled trials, as well as interrupted time-series analyses. We also considered observational analytical studies, including cohort, case–control and cross-sectional designs. Descriptive observational studies, such as case reports, case series and descriptive cross-sectional studies, were included. Qualitative research designs included methodologies such as phenomenology, grounded theory, ethnography, qualitative descriptive studies and action research. Systematic reviews that met the inclusion criteria were also incorporated. Opinion papers were excluded, whereas reflection articles were retained. Reflection articles were included because they usually offer structured, experience-based insights grounded in professional or empirical contexts, which can inform conceptual understanding. In contrast, opinion papers were excluded due to their predominantly subjective nature and lack of a methodological or experiential foundation.

Publications without full-text availability or extractable data (e.g., conference abstracts or letters to the editor) were excluded. Only studies published in Portuguese, English, or Spanish were considered. The eligibility criteria were applied consistently across all databases to guide the inclusion of gray literature by specifying the types of unpublished documents considered.

### 2.3. Information Sources

The research strategy aimed to identify both published and unpublished studies. An initial search was conducted using the Medical Literature Analysis and Retrieval System Online (MEDLINE), via PubMed, and the Cumulative Index to Nursing and Allied Health Literature (CINAHL), via EBSCO, to identify relevant articles on the subject. The words in the titles and abstracts of the selected articles, along with the indexing terms used to describe them, were analyzed to develop a comprehensive search strategy for relevant databases and information sources.

### 2.4. Search

The following databases were systematically searched: MEDLINE (via PubMed), CINAHL Complete (via EBSCO), the Web of Science Core Collection (via Web of Science), the ProQuest Dissertations & Theses Citation Index (via Web of Science), and Scopus. An experienced librarian collaborated on developing the search strategy, which included identifying relevant databases, refining search terms, and optimizing the overall search process.

During the development phase, several alternative search strategies were tested, including the use of broader and related terms, to ensure comprehensive coverage of the topic. The final strategy was selected because it provided the most appropriate balance of sensitivity and specificity for retrieving relevant studies. It combined controlled vocabulary (e.g., MeSH terms) and free-text terms related to children, nursing, and HL. It was also tailored to each database to account for differences in indexing systems and functionalities. This approach guaranteed the inclusion of all relevant keywords and subject headings.

The search was conducted on 25 July 2025, using search terms that were selected according to the chosen database. To illustrate a search strategy, we present the search equation used in the CINAHL Complete database: ((infan* OR toddler* OR child* OR teen* OR adolescen* OR pediatric* OR paediatric* OR youth OR schoolchild* OR preteen* OR “pre-teen” OR “young people” OR school* OR preschool*) OR MH (infant OR child OR “child, preschool” OR adolescence OR pediatrics OR schools)) AND (XB nurs* OR MH (nurses+ OR “nursing care+” OR “pediatric nursing”)) AND (XB (“health literacy” OR literacy) OR MH (“health literacy” OR literacy)).

The complete search strategy for each database, including the terms used and the search equation, is available on the scoping review registration page on OSF (https://osf.io/zv53s/, accessed on 25 August 2025).

### 2.5. Selection of Sources of Evidence

A pilot screening of a sample of studies was performed to ensure consistency across reviewers. After that, the researchers independently identified studies from the selected databases and imported them into Rayyan^®^ software, version 1.6.3 [19], removing duplicate articles in the process.

The researchers screened the articles, selecting them based on their titles and abstracts, and included or excluded them according to the inclusion and exclusion criteria of this scoping review. Next, the full texts of the selected articles were retrieved and read for analysis and selection. Two investigators independently reviewed the full texts of the studies to determine whether they met the inclusion criteria. If disagreements occurred, the articles were discussed among the reviewers. If consensus could not be reached, a third reviewer was consulted to make the final decision.

### 2.6. Data-Charting Process

Data-charting tables were developed as standardized instruments to guide the extraction of relevant variables. The data recording form was tested on a sample of studies and refined iteratively. Two reviewers conducted the data extraction independently (C.F. and M.S.) and subsequently compared their results to identify discrepancies. In cases of disagreement, a third reviewer was consulted to resolve inconsistencies and reach consensus. This process improved the accuracy and consistency of the extracted data.

### 2.7. Data Items

We extracted data on the characteristics of each article, including the author, year of publication, title, country, type of study, study objective, and assessment/instruments used. We then entered these data into a table (Supplemental File S2—Table S1). Since our focus was on interventions that promote HL, we created a second table describing the participants, including their age, characteristics, and sample size, as well as the nursing intervention, including its name, theme, duration/frequency, methodology, procedures, activities, materials used, health professionals involved, and context (Supplemental File S2—Table S2).

### 2.8. Critical Appraisal of Individual Sources of Evidence

In line with JBI guidelines for scoping reviews, which emphasize mapping the scope of available evidence rather than assessing the quality of individual studies, we did not critically appraise the included sources. This approach aligns with the exploratory nature of scoping reviews, the main objective of which is to identify key concepts, types of evidence, and research gaps within a specific field. By not restricting inclusion based on methodological quality, we were able to capture a comprehensive overview of the existing literature and provide a broad understanding of the topic under investigation.

### 2.9. Synthesis of Results

After screening, the extracted evidence was systematically organized into data-charting tables to facilitate comparison across studies. The data were then subjected to descriptive qualitative thematic analysis.

An inductive approach was adopted, whereby the coding was guided by the data rather than by predefined categories. First, the extracted data were carefully reviewed and open-coded to identify information relevant to the research objective. The codes were then systematically compared and grouped into broader categories based on similarities and relationships.

Through an iterative and reflexive process, the authors refined and synthesized the categories further to create overarching themes that captured the key characteristics, patterns and variations across the included studies. Throughout the analysis, the authors repeatedly revisited the data to ensure that their interpretations were evidence-based, thereby enhancing the depth and consistency of the findings.

This analytical process involved continuously organizing and reorganizing the data, identifying connections between concepts and developing thematic structures that reflected the nature, scope and diversity of the identified interventions. The research team regularly discussed coding decisions, refined thematic categories, and ensured coherent interpretation of the data. Any discrepancies or differences in interpretation were resolved by consensus through discussion, thereby strengthening the analysis’s credibility and reliability.

Initially, each author performed data extraction independently using a standardized approach. Subsequently, the research team collaboratively reviewed and validated the data extraction process to enhance the reliability, accuracy, and transparency of the synthesis process.

## 3. Results

### 3.1. Selection of Sources of Evidence

From the initial sample of 3338 articles, 107 were fully evaluated after applying the inclusion and exclusion criteria. Ultimately, we identified 44 articles and excluded 63. Data from the eligible articles were extracted using tables developed by the researchers and presented in narrative and tabular formats. Additionally, relevant data were extracted and analyzed using descriptive qualitative content analysis to address the scoping review’s objective.

**Figure 1:**
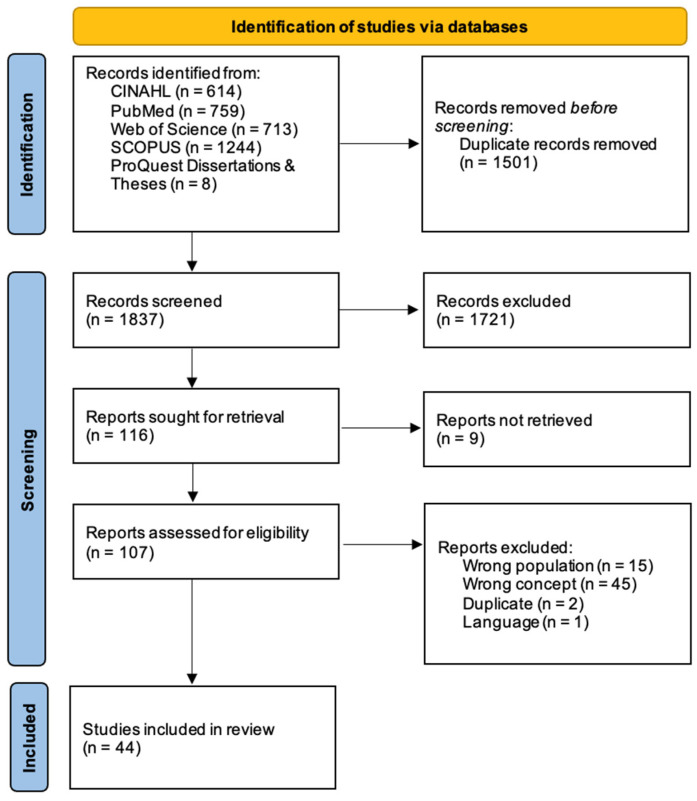
PRISMA-ScR flow chart for study selection.

Figure 1 shows the flowchart developed based on the model suggested in the PRISMA-ScR [20] guidelines. This flowchart ensures a structured presentation of the process of identifying, screening, selecting, evaluating, and synthesizing relevant studies.

### 3.2. Characteristics of Sources of Evidence

The study designs were diverse, including qualitative, quantitative, and mixed methods approaches. The content of the interventions included in the 44 studies clustered around a limited number of thematic areas. Nursing interventions were grouped into three domains: (1) health care, focusing on the self-management of chronic diseases; (2) health promotion, through nutrition, physical activity and mental health programs; and (3) disease prevention, including sex education and substance use prevention.

Themes addressed. The most frequently addressed topic was chronic disease management (n = 12), covering conditions such as asthma [21,22,23,24], cystic fibrosis [25,26], cancer [27,28,29], type 1 diabetes mellitus [30], epilepsy [31], and congenital heart disease [32]. The next most common themes were mental health (n = 7) [33,34,35,36,37,38,39] and nutrition/food-related literacy (n = 5) [30,40,41,42,43]. Emergent literacy (n = 2) [44,45], human papillomavirus (n = 2) [46,47], and substance use (tobacco, alcohol, and drugs) prevention (n = 2) [48,49] were addressed less frequently. Topics addressed by a single study included emotional literacy [38], perioperative preparation [50], and over-the-counter medication [51]. A smaller set of studies (n = 6) integrated multiple themes within broader HL programs [42,52,53,54,55,56].

Participants and context. There were studies (n = 7) in which the target population included young adults in addition to the pediatric population [22,28,29,33,38,42,55]. These studies were retained because adolescents were explicitly included in the target population, the interventions were directly relevant to adolescent HL, and the reported mean age of participants did not exceed 18 years. However, the findings were not reported separately from those of young adults. Therefore, these studies should be interpreted cautiously as representing transitional adolescent and young adult populations. Analysis of these articles reveals that nursing interventions mainly focused on adolescents, especially those aged 13 to 18 [28,35,37,55,57,58,59,60]. The findings also emphasize HL promotion in high school settings. While most studies prioritized this age group (adolescents), interventions aimed at school-age and preschool children were also identified. Studies with participants below preschool age were scarce [44,45].

Some of the interventions identified in the studies were directed not only at children and adolescents, but also at other participants, including parents, caregivers, and teachers [52,55]. However, they were included if the focus was on the nursing interventions directly applied by nurses to children and/or adolescents. During the selection process, it was confirmed that three articles reported on interventions carried out by nursing students [41,57,61]. Since these interventions were planned, supervised, or structured by nurses, they were considered relevant to the present scoping review because they reflect nursing practice.

Some articles [21,22,24,26,28,29,31,32,33,36,38,42,44,45,47,49,51,55,56,58,59,60,62,63] did not list nurses as the only individuals responsible for interventions. However, they were included since the articles describe nurses as part of a multidisciplinary team, and each professional’s work is important for promoting HL among children and adolescents.

Summary of nurse-led versus multidisciplinary interventions. Across the 44 included studies, interventions were nurse-led in 19 articles (including 3 delivered by nursing students under registered nurse supervision), and in 25 studies, interventions were delivered by multidisciplinary teams with a clearly identifiable, central nursing role. This summary clarifies how the interventions were classified, improving transparency when interpreting the evidence within this review.

The school environment was the most evident context of the intervention, and this context was described in most articles (n = 30). Five of these articles reported an exclusive online format [31,35,38,42,43]. Some of these articles described this adaptation as being due to the SARS-CoV-2 pandemic.

The community environment was mentioned in 10 articles [21,22,23,24,28,32,42,49,58,59]. These articles included events such as children’s parties, summer camps, church gatherings, and youth clubs, as well as an online format. Primary health care, namely health centers and pediatric clinics, was the context of intervention in nine articles [22,24,25,32,44,45,49,62,63]. The hospital was mentioned in six articles as a place of intervention [21,26,27,29,30,50]. One article stood out for taking place in a juvenile detention center [57].

### 3.3. Results of Individual Sources of Evidence

We extracted relevant data from each retrieved article and summarized it in Tables S1 and S2, which are presented as Supplemental Files along with this manuscript. Additionally, since one purpose of this scoping review was to identify nursing interventions that promote HL, a synthesizing table was constructed to consolidate and organize the key interventions that were identified across the included studies. To better delineate nursing’s contribution to multidisciplinary interventions, the identified strategies were mapped according to the NIC [64]. This process identified nursing-specific actions and roles within broader healthcare approaches. The resulting synthesis is presented in Table 1. This summary provides a quick and thorough understanding of the relationships between age groups, implementation contexts, and interventions.

In line with the aims of a scoping review, the table highlights the diversity of intervention types, activities and contexts in which nursing contributes.

**Figure 2:**
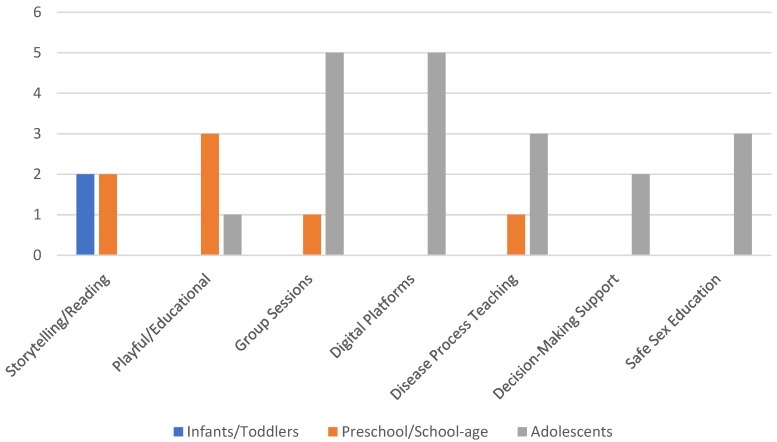
Distribution of nurse-led HL strategies by age group.

Figure 2 provides a visual representation of how nurse-led strategies are distributed across different developmental stages.

The graphic highlights the predominance of school-based strategies for adolescents and the use of playful and narrative strategies for younger children. However, variations in study designs and reporting limited the consistent inclusion of data on outcomes, highlighting important areas for future research.

### 3.4. Synthesis of Results

We identified studies from the United States [21,25,32,41,44,45,49,51,57,58,59,61], Turkey [31,35,36,40,43,53,54], Portugal [27,34,37,39,48,65], the United Kingdom [22,23,24,38], and Sweden [52,62], as well as other countries, including Australia [28], Canada [29], Egypt [30], Norway [33], Nepal [55], Brazil [50], Germany [56], Taiwan [60], New Zealand [47], and the Dominican Republic [42]. These studies were published between 1997 and 2025, with 66% published between 2020 and 2025.

Despite variability in study design, the synthesis was primarily based on research evidence because most of the studies were primary empirical studies (n = 36). Beyond thematic frequencies, the included evidence shows consistent, cross-study patterns linking developmental stage, delivery format, and implementation context. Interventions targeting infants and toddlers were rare (n = 2) and clinic-based [44,45]. These interventions focused on emergent literacy through shared reading and book distribution during routine child health visits. In contrast, most interventions focused on adolescents and frequently employed participatory group education methods (e.g., dialogue, debate, and skills-building). Several studies [22,35,43,48,51,60] used digital delivery formats (e.g., web-based modules, apps, or messaging platforms). Context and topic also covaried. School settings predominated across the evidence base, positioning schools as the main arena for HL, particularly in mental health [33,34,35,36,37,38,39] and nutrition-related [40,41,43,59,60,61] interventions. Condition-specific self-management programs (e.g., asthma, diabetes, epilepsy, and cancer) were more commonly linked to healthcare contexts (e.g., hospitals, clinics, and primary care), where teaching activities by nurses focused on disease processes, treatment adherence, and symptom monitoring.

## 4. Discussion

The promotion of HL among children and adolescents is an essential strategy to promote autonomy in informed decision-making throughout life. Our findings suggest that nursing interventions to promote HL are shaped by the interaction between developmental stage, setting, delivery format, and HL domain.

For younger children, HL was mainly promoted through concrete, playful, and narrative strategies that translated health-related information into accessible experiences. Among adolescents, interventions shifted toward more participatory, reflective, and digitally mediated approaches, which is consistent with their growing autonomy, critical thinking abilities, and involvement in health-related decision-making. Similarly, school-based interventions were predominantly linked to health promotion and disease prevention, whereas clinical and primary care settings were more closely associated with the healthcare domain, particularly disease-specific education and self-management. These patterns suggest that nursing interventions do not promote HL through a single approach, but rather adapt their strategies according to age, context, and the type of health-related competence being targeted.

The concept of HL was not consistently defined across the included studies, reflecting the coexistence of multiple theoretical frameworks and operational approaches within the broader field of HL. Some studies adopted comprehensive definitions encompassing functional, interactive, and critical dimensions, while others focused more narrowly on basic skills, such as reading and understanding health information. This variability also extended to the instruments and indicators used to measure HL, which further contributed to differences in how the concept was interpreted and applied.

This variability may limit the comparability and interpretability of the findings because differences in conceptualization can affect study design and the outcomes reported. It may also hinder the ability to draw clear conclusions or identify consistent patterns in the existing literature. These challenges highlight the need for improving conceptual clarity and standardization in future research. This includes adopting well-defined, theoretically grounded frameworks to enhance consistency and support more robust evidence synthesis.

Examining the identified interventions through a theoretical lens revealed that they predominantly targeted functional HL, emphasizing improving children’s ability to access and understand health information. A smaller number of interventions extended into the interactive domain by fostering communication skills, self-efficacy, and engagement in healthcare interactions. Fewer studies addressed critical HL, such as developing appraisal skills, critical thinking, and the ability to question and apply health information in broader social contexts. This uneven distribution highlights a tendency for pediatric HL interventions to prioritize foundational competencies over advanced, empowerment-oriented dimensions.

From a complementary perspective, the interventions can be situated within the three domains proposed by Sørensen et al. [3]: healthcare, disease prevention, and health promotion. Most interventions were located within the healthcare and disease prevention domains and focused on supporting children’s understanding of conditions, treatments, and risk factors. Those aligned with health promotion were less frequent but were more closely associated with interactive and critical HL dimensions. This was particularly true when the interventions encouraged autonomy, informed decision-making, and active participation in health-related behaviors.

These findings suggest that existing nurse-led HL interventions meaningfully contribute to the development of pediatric HL; however, they remain largely concentrated at the functional level. This indicates an important opportunity to advance the field by designing and implementing interventions that deliberately integrate interactive and critical dimensions. This shift could lead to a more comprehensive, developmentally appropriate conceptualization of pediatric HL that emphasizes understanding, engagement, reflection, and empowerment in health-related decision-making.

The analysis of the results highlights the advances and gaps in each domain. In the field of health care, the mapping of interventions for chronic diseases stands out. School and digital programs have shown a predominance in health promotion. In disease prevention, there is a need to expand interventions for vulnerable contexts. While these findings are consistent with previous reviews that emphasise the predominance of adolescent-focused interventions, they also extend the literature by emphasizing the role of nursing-led strategies.

In addition, an unequal representation of socio-cultural contexts was identified, with limited consideration given to vulnerable populations such as rural communities and ethnic minority groups. In view of these challenges, this section seeks to synthesize the available evidence, propose ways to overcome the identified weaknesses, and reinforce the strategic role of nurses in promoting HL from childhood.

Although this review mapped relevant nurse-led interventions to promote HL, it is essential to recognize that most included studies were conducted in Western countries, particularly in school-based settings. This geographic and organizational concentration introduces important limitations regarding the transferability of findings to other cultural, economic, and institutional contexts.

The predominance of school-based interventions assumes the existence of structured educational systems and regular nurse presence in schools. However, this may not reflect the reality in low-income countries, rural areas, or communities with limited resources. Furthermore, cultural values, family practices, and health beliefs vary widely across regions, potentially influencing both the acceptance and effectiveness of interventions. Strategies that have been proven effective in Western contexts may need to be significantly adapted to be relevant and feasible elsewhere.

The scarcity of studies involving vulnerable populations, ethnic minorities, and non-school settings limits the generalizability of the findings. This highlights the need for future research to prioritize the cultural adaptation of interventions, the inclusion of diverse organizational contexts (such as community, primary care, and informal environments), and the active participation of underrepresented groups in the design and evaluation of strategies.

Therefore, future investigations should explicitly consider the cultural, economic, and organizational specificities of target contexts, promote co-construction of interventions with local communities, and rigorously assess their transferability and impact. Only through these approaches can the reach and effectiveness of nurse-led HL interventions for children and adolescents be expanded globally.

The implications for practice and research are also discussed. The focus is on developing innovative, inclusive and sustainable interventions that consider the specific needs of each age group and sociocultural context.

### 4.1. Nature and Characteristics of Interventions

The interventions promoting HL included a wide range of presentation formats and strategies.

Among infants and toddlers, only two studies targeted these age groups. Both studies referred to the ROAR program, which was developed in the United States [44,45]. From an early age, the importance of books and reading was emphasized during routine child health surveillance consultations. These interventions have “the potential to improve children’s early literacy development. This is particularly important for at-risk households where parents may have poor literacy skills and limited financial resources” [45]. In addition to its importance for HL, it is worth noting that the playful and participatory nature of this intervention allowed children to view health contexts differently, making them feel more familiar and safer [66]. These findings suggest that early-life interventions lay the groundwork for HL by promoting language development, parent–child interaction, and familiarity with healthcare environments, especially among families facing socioeconomic challenges.

For preschoolers and school-age children, strategies that take into account their cognitive development are preferred, such as storytelling [23,24], books [26,41,44,45,59], games (both board and digital) [23,24,32,33,48,54,55,56,61], songs [23,24], educational videos [24,31,32,36,37,41], and interactive activities like role-playing [23,34,55,56,57,58]. Children find illustrations, comics, and books attractive and vehicles for health information and knowledge [50]. Storytelling can encourage cooperative behavior and teach children responsibility [24]. In this sense, it was interesting to find books that children and young people know, such as the Harry Potter series, which can help them understand certain concepts about genetics, such as those related to cystic fibrosis [25]. Another example is the book The Three Little Pigs, which retells the classic story while promoting asthma awareness and proper inhaler use [24]. The use of books as an important strategy and resource for health professionals to promote HL is well established in the literature [66]. Most of these interventions took place during child health surveillance consultations in clinics or health centers and in schools. They were integrated into the children’s pre-existing routines to improve adherence. The mapped interventions suggest that developmentally appropriate, engaging strategies are commonly used to support children’s understanding of health information and encourage their participation in the learning process. These approaches appear to promote early HL by encouraging interaction, autonomy and meaningful engagement in familiar contexts.

For adolescents, the interventions included weekly group sessions that used participatory methodologies, such as dialogue and debate, to promote critical reflection, as well as the development of practical skills [30,51,57] related to mental health, diet, and healthy lifestyles. These educational sessions could be individual [48,58] or group sessions [22,40,58] and varied in frequency and duration (from single sessions to multi-week programs). Other interventions included workshops [61], role-playing [23,34,55,56], art [43], peer mediation [56], videos [36,37,49], games [33,54,58], group discussions [36,49], and stories [24,55] focusing on healthy behaviors. Digital platforms [28,31,32,38,48] were widely used by this age group, providing young people with access to informative and interactive content on topics such as mental health, nutrition, physical exercise, and chronic disease management (e.g., mobile applications for medication and symptom monitoring and control). Access to these platforms varied in duration (e.g., one study provided access to a free app for two months) [28]. Online interventions incorporated educational activities, quizzes, videos, and discussion forums, offering opportunities to interact with field-specific professionals. The importance of discussions and reflections on transmitted content among peers (adolescents) and between them and health professionals is emphasized. The identified interventions suggest that participatory and socially interactive approaches are commonly used to promote HL among adolescents.

The predominance of interventions focused on adolescents may reflect developmental factors, such as their greater capacity for critical engagement and autonomous learning. It may also reflect the alignment of HL initiatives with age-specific challenges, such as mental health issues, risky behaviors, and self-management. This idea is supported in the recent literature, as evidenced by articles by Mancone and colleagues [67] and Asplund et al. [68]. The concentration of interventions in school settings likely results from schools’ accessibility, structured environments, and ability to integrate HL into existing curricula [69]. This facilitates consistent implementation and peer-based participatory approaches.

The mapped interventions suggest that variations in nursing approaches to HL are associated with both the topic addressed and the alignment between developmental stage, delivery format and setting. Interventions for younger children incorporated symbolic, sensory and narrative resources to translate health information into concrete, familiar experiences. In contrast, interventions targeting adolescents more frequently involved participatory and digitally mediated formats, reflecting their growing independence and critical thinking abilities. The setting also appeared to influence the focus of interventions: school-based initiatives were often oriented towards universal and preventive approaches, while those implemented in clinical contexts tended to emphasize personalized education related to disease self-management. Overall, these patterns emphasize the importance of designing HL interventions that are developmentally appropriate, context-sensitive and aligned with the specific HL domains being addressed.

### 4.2. Interventions Domains

The interventions can be categorized into three main domains, as conceptualized by Sørensen et al. [3]: healthcare, disease prevention, and health promotion.

Interventions targeting children and adolescents that are mapped tend to address HL alongside the promotion of healthy behaviors. Programs focusing on managing chronic diseases, such as asthma [23,24], diabetes [30], and epilepsy [31], appear to increase knowledge and encourage self-management while reducing hospitalizations and absences from school. Digital resources and playful methodologies appear to contribute to learning and reducing disease-related anxiety, emphasizing the role of nursing professionals in education and treatment adherence [38]. Another key focus is prevention, with strategies aimed at substance use [31,49] and sexual and reproductive health [46,47,57,63,65]. These initiatives apparently increase knowledge of risks, strengthen decision-making skills, and encourage preventive behaviors, even among vulnerable groups. Integrating these strategies into the school curriculum and involving trained professionals can promote positive changes in the attitudes and habits of children and adolescents [69]. The identified interventions suggest that HL strategies often encompass knowledge development alongside skills such as decision-making, self-management and engagement with care. These findings reflect the multidimensional nature of HL and its potential relevance in pediatric contexts.

In the context of disease prevention, nursing strategies focused on topics such as sexual and reproductive health, as well as the prevention of sexually transmitted infections among young people [46,47,57,63,65]. These initiatives included young people from various backgrounds, particularly those from socially vulnerable contexts, such as juvenile detention centers [57]. Often integrated into school curricula, these initiatives are described in the literature as having the ability to promote knowledge of anatomy, sexually transmitted infections, contraception, and risky behaviors [57]. The mention of HL as a protective factor was consistent across the different studies, which apparently not only increased knowledge but also enhanced changes in attitude and behavior. In the article by Watson and Serrant-Green [46], the importance of HL among young girls regarding human papillomavirus is emphasized, and the authors report that the intervention significantly improved outcomes for a group of young people aged 12–13. This demonstrates the effectiveness of the educational session designed for adolescents. These findings reinforce the importance of HL in disease prevention.

In a school setting, health promotion initiatives focusing on nutrition [40,59,60,61], physical activity [40,43], and mental health [33,34,35,36,37,38] are essential. Using participatory methodologies, adapted content, and digital technologies makes information more accessible and encourages the adoption of healthy habits. Programs such as cooking workshops, gardening, and hands-on activities appear to increase young people’s autonomy. Meanwhile, mental health interventions promote recognizing and managing emotions, apparently reduce stigma, and strengthen socio-emotional skills [33,34,35,36,37,38]. These findings underscore the importance of HL in promoting health by improving adolescents’ ability to access, understand, and apply health information. This, in turn, appears to support the development of autonomous, health-promoting behaviors.

The mapped studies identified nursing interventions across all categories. However, the implementation of pediatric HL interventions may be hindered by limited resources and time, difficulties in interdisciplinary collaboration, and inadequate cultural and linguistic adaptation. Furthermore, the complexity of assessing HL in children underscores the necessity of more robust, context-sensitive evaluation strategies.

### 4.3. HL Evaluation Instruments

There is significant variability in the instruments and methodologies used to assess HL. Only a few studies used validated, specific instruments, such as the Mental Health Literacy Questionnaire (MHLQ)-Adolescent Version (A-MHLQ) [37,39], the Health Literacy Scale for School-Age Children [40,53], the Epilepsy Health Literacy Scale (EHLS) for adolescents [31], and the e-Health Literacy Scale (eHEALS) [31]. Using adequate and validated instruments is essential to accurately identify the target population and ensure the validity and reliability of the collected data collected [70,71].

Since few instruments have been validated for assessing HL, especially among younger age groups, some studies have used their own questionnaires or validated health promotion questionnaires. Examples include the Healthy Nutrition and Physical Activity Self-Efficacy Scale for Children [40], the Adolescent Health Promotion Scale [40,43,54], and the Epilepsy Knowledge Questionnaire (EKQ) [31]. One of the included studies used the Positive Mental Health Questionnaire (QSM+) [37] as an assessment tool. Qualitative and exploratory studies favored methodologies such as interviews and focus groups because they are useful for gaining a deeper understanding of participants’ perceptions and experiences. However, they are more difficult to replicate.

The absence of standardized and/or validated instruments for different age groups is an important limitation and challenge for future investigations. Several of the analyzed studies [32,36,55,56] also identified the lack of long-term evaluations as a challenge. The included studies demonstrate substantial heterogeneity in HL assessment methods, with limited use of standardized, validated, developmentally appropriate instruments. This variability complicates comparisons across studies and limits clarity regarding the constructs being measured. This issue is particularly relevant in pediatric populations due to developmental differences. Thus, the diversity of assessment approaches is a central finding of this scoping review. This measurement gap may compromise the ability to compare, evaluate, replicate, and translate nursing interventions into practice.

### 4.4. Implication for Practice

Nurses have the autonomy to design and implement HL interventions for children and adolescents. This underscores the profession’s proactive and leadership role in promoting health from an early age, as well as its responsibility to individuals and society. However, since this scoping review did not evaluate the methodological quality or risk of bias of the included studies, the implications for practice should be considered preliminary and evidence-based rather than definitive.

Although progress has been made in recent years, important challenges remain, particularly within political and educational systems. Although integrating HL into nursing education and training has shown promise in improving professional competencies [10], further research is needed to evaluate its long-term impact on clinical practice and patient outcomes.

Despite the existence of the National Health Promoting Schools Program, the findings also suggest that integrating HL-oriented activities into school curricula, beginning in primary education, could be considered a promising area for practice and policy development. This approach has been successful in countries such as Sweden and Norway and includes Health Dialogues [52]. In addition to this program, projects like ROAR, which promote emergent literacy among children, should be considered.

The regular presence of nurses in schools would also be valuable. Almost all of the studies included in this scoping review reinforce this idea, but further research is needed to determine the effectiveness and feasibility of this approach.

The included studies used different definitions of HL, and most interventions were not clearly described in terms of the functional, interactive, or critical levels. Therefore, a systematic classification based on these dimensions could not be performed because it would have required assumptions beyond the reported data. Future studies would benefit from clearer descriptions of the HL dimensions addressed by interventions.

In summary, robust, collaborative, and continuous research is necessary to establish HL as a fundamental pillar for the healthy development of children and adolescents and to promote effective, lasting health gains for future generations.

### 4.5. Implications for Research

The inconsistent research design, the scarcity of interventions implemented in younger age groups, and the lack of validated HL assessment tools for different age ranges among the 44 analyzed studies highlight the need for future research.

Despite progress in designing and implementing nursing interventions to promote HL in children and adolescents, significant gaps remain. Notably, there is a need to develop and validate age-appropriate tools to assess HL across different developmental stages. This is particularly important for children under 10 years old because most existing instruments are tailored for adolescents or adults. Addressing this gap is a key research priority. Future studies should clearly define the HL construct being assessed and indicate which dimension is targeted (functional, interactive, or critical). They should also use developmentally appropriate measurement instruments. Standardizing the assessment of HL would enhance comparability and facilitate the replication of studies, ultimately strengthening the evidence base for nursing interventions that promote HL. Additionally, longitudinal studies are recommended to evaluate the medium- and long-term impact of interventions. These studies would improve our understanding of the sustainability of HL gains and their impact on children’s and adolescents’ behaviors and decision-making processes. Future research should also examine the effects of interventions in diverse contexts, such as among vulnerable populations, in rural areas, and within ethnic minority groups.

There is a scarcity of directly applied nursing interventions for younger age groups as well. This reinforces the prevailing assumptions about children’s ability to actively participate [72]. Further research is necessary to evaluate the implementation of interventions at an earlier age, as preschool-aged children have the cognitive capacity to benefit from them. Developing HL from early childhood may improve health behaviors and outcomes. Therefore, it is crucial to identify the most effective strategies to support children’s HL and to develop effective ways to monitor their progress throughout their life cycle. Furthermore, the integration of digital technologies and innovative methodologies into intervention design should be further explored to value the active participation of children and adolescents in the educational process.

To synthesize our main findings, we used the framework proposed by Bradbury-Jones et al. [73] that considers the synthesis into five topics: Patterns, Advances, Gaps, Evidence for Practice and Research Recommendations (PAGER) as detailed in Table 2.

Although this scoping review focused on mapping existing interventions, considerations related to implementation, such as resource availability, scalability and contextual adaptability, remain underreported. This highlights the need for future research to provide a clearer picture of how these interventions can be translated into practice.

In line with the JBI methodology, this scoping review did not include a critical appraisal of the studies included. While this approach allows for a comprehensive mapping of the available evidence, it limits the ability to assess the included studies’ methodological quality and risk of bias. Consequently, the conclusions drawn from this review should be interpreted with caution. Future systematic reviews with rigorous critical appraisal are recommended to strengthen the evidence base. The recommendations provided in this scoping review are based on the synthesis of the available evidence, which is limited by the heterogeneity of study designs and the absence of standardized measurement tools. Therefore, these recommendations should be interpreted as preliminary and subject to further validation through rigorous empirical research.

The results of this scoping review highlight a set of gaps that have significant implications for both health policy development and clinical nursing practice. The absence of validated, appropriate tools for assessing child development, particularly for children under ten years of age, hinders health systems’ ability to systematically and comparably monitor HL. Without robust metrics, it is difficult to define priorities, guide investments, and evaluate the effectiveness of HL promotion programs. This limitation also has repercussions in clinical practice, where nurses lack the tools that allow them to identify specific needs and adapt educational interventions precisely, which compromises personalized care.

Another critical barrier is the scarcity of longitudinal studies. The absence of evidence on the medium- and long-term effects of nursing interventions prevents policymakers from recognizing child HL as a structuring investment with a return throughout the life cycle. Consequently, policies to promote HL tend to be fragmented, episodic, and poorly sustained. In clinical practice, this gap translates into the difficulty in selecting strategies with proven efficacy, which limits nurses’ ability to base their choices on robust evidence.

Another emerging challenge is the limited evidence on integrating digital platforms with participatory methodologies. While digital technologies have the potential to expand the scope of interventions, the lack of validated hybrid models makes it difficult to create policies that promote the safe, ethical, and effective use of these tools. In clinical practice, nurses can adopt digital resources without a guarantee of pedagogical adequacy, which compromises the quality of interventions.

Finally, the lack of methodological standardization compromises the comparability between studies and makes it difficult to carry out meta-analyses that could support more robust policy recommendations. The lack of consistent outcome measures and harmonized reporting frameworks weakens the available evidence and limits its applicability. In clinical practice, this heterogeneity makes it difficult to assess the impact of interventions and build evidence-based protocols.

### 4.6. Limitations

Despite efforts to ensure the comprehensiveness and methodological rigor of this scoping review, several limitations must be acknowledged. First, restricting the search to studies that directly targeted children with HL interventions may have resulted in the exclusion of relevant studies addressing strategies aimed at parents, caregivers, or other intermediaries with the same purpose. The heterogeneity of the included studies, ranging from qualitative and quantitative designs to systematic reviews and randomized controlled trials, limited the use of standardized methodological quality assessment tools and prevented direct comparison of results and quantitative syntheses. The study selection process, which was based on predefined inclusion criteria and the availability of data in the consulted databases, may have also resulted in the exclusion of relevant studies.

All included interventions involved a clearly identifiable nursing contribution; the extent of nurses’ participation varied across studies, ranging from intervention leadership to participation within multidisciplinary teams. The level of detail reported regarding professional roles was inconsistent, which may limit the precise characterization of nursing involvement in some interventions.

Evidence was distributed unequally across age groups and sociocultural contexts. Adolescents were disproportionately represented, whereas interventions targeting infants, toddlers, and preschool-aged children were scarce. Similarly, ethnic minority groups, rural populations, and other socially vulnerable communities were insufficiently represented, restricting the generalizability of the findings.

Another limitation concerns the inclusion of studies with mixed adolescent and young adult samples. Although these studies were retained because adolescents were included and the mean age did not exceed 18 years, it was not possible to isolate pediatric outcomes in all cases, and these findings should therefore be interpreted with caution.

Finally, the inconsistent definitions of HL and its domains across studies may have limited the consistency of the reviewed concepts and evaluation indicators. The focus on explicit HL terminology supported conceptual consistency but may have limited search sensitivity, potentially excluding relevant interventions described using broader or related terms. Despite these limitations, this scoping review provides a comprehensive and up-to-date overview of nursing interventions aimed at promoting HL in children and adolescents.

## 5. Conclusions

The results of the studies included in this review link nursing interventions to improvements in HL levels, increased autonomy, and more informed decision-making by participants. The interventions covered different age groups, with an emphasis on adolescents. Overall, the interventions stood out for their adaptation to context and stage of development, using methodologies that favor the active participation of children and young people, as well as playful approaches.

Among adolescents, interventions based on digital platforms and group sessions with participatory methodologies were identified. These interventions were often focused on mental health, nutrition and healthy lifestyles. The strategies for preschool and school-age children were adjusted to cognitive development, favoring narratives, books, games, music, and interactive activities. Only two studies included younger populations, such as infants and young children.

However, it is important to emphasize that, although the literature reveals positive results associated with nursing interventions, the objective of this study was to map and characterize these interventions rather than evaluate their effectiveness. Thus, the conclusions should be interpreted within this exploratory framework. They should reflect the trends and patterns identified in the included studies without making impact judgments that go beyond the scope of the review. This methodological alignment strengthens the internal consistency of the work, maintains the conceptual integrity of the scoping review, and facilitates the clear communication of its contributions to the field of HL and nursing interventions.

In light of the identified gaps, clear priorities must be established to guide the field’s strategic and coherent advancement. This review highlights three priorities: first, developing validated and standardized instruments to assess HL; second, implementing longitudinal studies to better understand how HL evolves over time; and third, systematically including younger populations, who are underrepresented in current research.

This review offers a thorough synthesis of nurse-led interventions aimed at promoting HL among children and adolescents, contributing to the field. The review emphasizes the importance of these interventions for nursing practice and research, underscoring the need for age-appropriate, context-sensitive strategies.

## Figures and Tables

**Table 1:** Nursing interventions promoting HL in children and adolescents, their activities, and implementation contexts identified in the included studies.

Intervention (NIC)	Activities Identified	Population/Context
Health Education (5510)	Group sessions on nutrition, physical activity, and healthy lifestyles; substance abuse prevention programs.	Adolescents (Schools and Health Centers)
Learning Facilitation (5520)	Use of participatory methodologies, dialogues, debates, and critical reflection on mental health.	Adolescents
Teaching: Disease Process (5602)	Education for self-management of chronic diseases such as asthma, type 1 diabetes, epilepsy, and cancer.	Children and Adolescents (Hospital and Clinics)
Cognitive Stimulation (4720)	Reach Out and Read (ROAR) program; promoting emergent literacy and early reading habits.	Infants and Toddlers
Bibliotherapy (4680)	Use of storybooks (e.g., “The Three Little Pigs” for asthma) and educational comic books.	Preschool and School-age Children
Play Therapy (4310)	Playful strategies, board games, digital games, puppet theater, and role-playing.	Children (Schools and Consultations)
Decision-Making Support (5250)	“Health Dialogues” to discuss well-being and facilitate informed health choices.	Students (School Setting)
Teaching: Safe Sex (5622)	Education on anatomy, contraception, and prevention of sexually transmitted infections.	Adolescents (including vulnerable contexts)

**Table 2:** Summary of the scoping review according to the PAGER framework.

Patterns	Advances	Gaps	Evidence for Practice	Research Recommendations
Interventions are consistently adapted to the cognitive stage of the participants. Younger children are mainly engaged through play, storytelling, books, games and visual resources, while adolescents are targeted through more participatory, group-based, digital and autonomy-oriented strategies.	The use of literary icons and creative narratives, such as using Harry Potter to explain genetics or The Three Little Pigs to teach asthma inhaler techniques, has made complex medical concepts accessible to children.	There is a significant scarcity of nursing interventions specifically targeted at children under 10 years old, with most research focused on the 13–18 age range.	Storytelling and play-based activities reduce disease-related anxiety and improve cooperative behavior during clinical consultations.	Future studies should focus on implementing and evaluating the effectiveness of interventions in earlier childhood (preschool) to capitalize on early cognitive capacity.
The school environment is the most prevalent context for promoting HL, serving as a “privileged” space for reaching large groups of children and adolescents.	The integration of digital technologies, including mobile apps for symptom monitoring and WhatsApp support groups, has improved health-related knowledge and engagement among adolescents.	There is a critical lack of standardized and validated measurement instruments to assess HL levels within the school setting, particularly for younger age groups.	Structured “Health Dialogues” between students and school nurses—already successful in Sweden and Norway—strengthen socio-emotional skills and mental well-being.	Establish official HL programs within the primary school curriculum and evaluate their impact through the development of age-appropriate assessment tools.
Nursing interventions frequently focus on healthcare, the management of chronic diseases and the prevention of risk behaviors.	Programs like ROAR have advanced early literacy by integrating book distribution and reading habits into routine pediatric surveillance starting at 6 months of age.	There is a lack of data on long-term effects, making it difficult to determine if HL gains in childhood translate into better health outcomes in adulthood.	Interventions in specialized settings, such as juvenile detention centers, were linked to improved sexual HL and greater engagement in preventive behaviors.	Prioritize longitudinal studies that evaluate the sustainability of HL gains and their impact on decision-making processes throughout the life cycle.

## Data Availability

No new data were created or analyzed in this study. Data sharing is not applicable to this article.

## Supplementary Materials

The following supporting information can be downloaded at: https://www.mdpi.com/article/10.3390/healthcare14131829/s1, Table S1: Data extraction table; Table S2: Key features of nursing intervention to promote health literacy in children and adolescents; File S1: PRISMA ScR Fillable Checklist; File S2: Tables.

## Abbreviations

The following abbreviations are used in this manuscript:

CIIS	Centro de Investigação Interdisciplinar em Saúde
CINAHL	Cumulative Index to Nursing and Allied Health Literature
EBSCO	Elton B. Stephens Company
FCSE	Faculdade de Ciências da Saúde e Enfermagem
HL	Health Literacy
JBI	Joanna Briggs Institute
MEDLINE	Medical Literature Analysis and Retrieval System Online
NIC	Nursing Intervention Classification
OSF	Open Science Framework
PCC	Population, Concept, Context
PRISMA-ScR	Preferred Reporting Items for Scoping Reviews
UCP	Universidade Católica Portuguesa
WHO	World Health Organization
