# Supplementary material for: Nursing Interventions to Promote Health Literacy in Children and Adolescents: A Scoping Review
Source: Healthcare (Basel). 2026 Jun 24;14(13):1829. doi: 10.3390/healthcare14131829 (PMC13361545; doi:10.3390/healthcare14131829)
Supplement: Supplementary file 1 [file healthcare-14-01829-s001.zip › Suplemmental file_S2_Tables.pdf]

Supplemental File 2

Table S1 - Data extraction table

Author, year of publication	Title	Country	Type of study	Purpose	Assessment / Instruments
Dülger *et al*., 2024	*The effect of health literacy-grounded web-based education on nutrition and exercise behaviours in adolescents: A randomized controlled trial*	Turkey	Randomized controlled clinical trial (experimental study)	To evaluate the effect of an online health literacy-based education program on health literacy levels, nutritional behaviors, and physical activity among adolescents.	*Health Literacy Scale for School-Aged Children*, *Nutrition and Exercise Subscales* da *Adolescent Health Promotion Scale*
Son *et al*., 2017	*The Effect of Interprofessional Student-Led Reproductive Health Education on Youths in Juvenile Detention*	United States of America	Prospective cohort study (quasi-experimental)	To evaluate the effects of a student-led interprofessional educational program on reproductive health literacy among youth in juvenile detention centers.	Pre- and post-intervention questionnaires: knowledge of reproductive health, behaviors, and risks; *Global Condom Use Self-Efficacy Scale* *Contraceptive Self-Efficacy Scale* *Sexual Self-Efficacy Scale*
Derwig *et al*., 2021	*Child-Centred Health Dialogue for primary prevention of obesity in Child Health Services – a feasibility study*	Sweden	Feasibility study with a quasi-experimental, non-randomized cluster design	To assess the feasibility of a child-centered dialogue model for the primary prevention of obesity among children receiving care at pediatric health services	Not applicable
Yoos *et al*., 1997	*An asthma management program for urban minority children*	United States of America	Descriptive study involving qualitative data collection (interviews and *focus groups*) and a literature review	To develop, implement, and evaluate a comprehensive asthma management program for urban children from ethnic minority groups, considering their family, social, and cultural contexts, and promoting health literacy and self-management of the disease.	Structured telephone interviews; *Focus groups* with children, adolescents, and family members

Author, year of publication	Title	Country	Type of study	Purpose	Assessment / Instruments
Almquist *et al*., 1998	*Bringing an early pediatric literacy program to the clinic setting*	United States of America	Case report / descriptive study on the implementation of an educational program in a pediatric clinical setting.	To describe the development and implementation of the Children's *Reach Out And Read* program, based on the national *Reach Out and Read* initiative, with the aim of promoting early literacy, emotional bonding, and cognitive development in children through shared reading between parents and children.	Quantitative questionnaire administered to parents (regarding reading habits, number of books at home, and library use); Observation and qualitative feedback from professionals, volunteers, and families
Atkinson *et al*., 2002	*Reach Out and Read: A pediatric clinic-based approach to early literacy promotion*	United States of America	Descriptive study and implementation of a program in a pediatric clinical setting.	To present and describe the "*Reach Out and Read*" program, which aims to promote early literacy in children by integrating literacy promotion into pediatric primary health care	Not applicable
Stonbraker *et al*., 2020	*Digital Educational Support Groups Administered through WhatsApp Messenger Improve Health-Related Knowledge and Health Behaviors of New Adolescent Mothers in the Dominican Republic: A Multi-Method Study*	Dominican Republic	Multi-method study	To assess whether participation in digital educational support groups via *WhatsApp* improves health knowledge, health behaviors (family planning, regular attendance at child health checkups), and the autonomy of adolescent mothers in a resource-limited setting.	*Health Literacy Screener*; Questionnaire on knowledge of maternal and child health; post-intervention interviews; Review of clinical records (frequency of visits, contraceptive use, and infant vaccinations)
Thomas, 2003	*Identifying and intervening with girls at risk for violence*	United States of America	Theoretical-reflective study based on empirical evidence and practical recommendations	To describe the profile of girls at risk of violent behavior and propose a nursing intervention model based on emotional literacy, parental education, and psychosocial support	Not applicable
Bereznay *et al*., 2019	*Promoting Food Literacy in Teens*	United States of America	Descriptive and Evidence-Based Practice Article - Proposal for an Educational	To promote food literacy among adolescents through health education led by pediatric nurses, with the aim of improving eating behaviors and preventing obesity	Not applicable

Author, year of publication	Title	Country	Type of study	Purpose	Assessment / Instruments
			Intervention in Pediatric Nursing.		
Kemble *et al*., 2024	*Children and young people's self-reported experiences of asthma and self-management nursing strategies: An integrative review*	United Kingdom	Integrative review	To explore the experiences of children and adolescents with asthma and identify effective nursing strategies for self-management of the condition, with implications for health literacy	Not applicable
Driessnack, 2009	*Growing up at the intersection of the genomic era and the information age*	United States of America	Case study	To illustrate and discuss the influence of children's learning contexts on their understanding of genetics and health, and how this prior understanding can be leveraged in educational interventions	Not applicable
Kuzma *et al*., 2024	*Re-envisioning Youth Sexual Health Care: Supporting Sex Positivity in a Digital World*	United States of America	Theoretical article and narrative review	To provide resources and information to help nurse specialists incorporate a *sex-positive* approach to young people's sexual health, including education on pornography, safe sexting, and consent, tailored to today's digital reality	Not applicable
Millar *et al*., 2024	*It takes a village to raise a child- A multidisciplinary approach to promoting paediatric health literacy in cystic fibrosis*	Ireland	Descriptive Study	To promote health literacy among children with cystic fibrosis through the collaborative development of age-appropriate, easy-to-read, and engaging educational materials; and to assess the impact of this intervention on the knowledge and engagement of children and their families	Not applicable
Akca & Ayaz-Alkaya, 2024	*Effectiveness of health literacy-based motivational interviewing and health education on adolescents' nutrition and exercise behaviors in Turkey: A randomized controlled trial*	Turkey	Randomized controlled clinical trial with two intervention groups (motivational interviewing and	To evaluate the effectiveness of health literacy-based motivational interviewing and health education in improving nutrition and physical activity behaviors among adolescents	*Health Literacy Scale for School-Age Children; Adolescent Health Promotion Scale; Healthy Nutrition and Physical Activity Self-Efficacy Scale for Children*

Author, year of publication	Title	Country	Type of study	Purpose	Assessment / Instruments
			health education) and a control group		
Watson & Serrant-Green, 2012	*Exploring HPV awareness and understanding before and after health education*	Reino Unido	Phenomenological pilot study	To assess the level of knowledge and understanding of HPV and the vaccine among female adolescents before and after a health education presentation conducted by school nurses, evaluating the impact of the educational intervention	Structured questionnaire administered before and after the educational intervention (unvalidated scale)
Kostenius *et al*., 2021	*Health Dialogue Experiences of Students, Teachers and School Nurses in Swedish Health-promoting Schools*	Sweden	Phenomenological study	To explore how students, teachers, and school nurses experience health-related dialogue in health-promoting schools, and how they contribute to the promotion of health literacy and school health.	Not applicable
Bjørnsen *et al*., 2018	*Exploring MEST: a new universal teaching strategy for school health services to promote positive mental health literacy and mental wellbeing among Norwegian adolescents*	Norway	Longitudinal cohort study	To investigate differences in the potential average outcomes regarding positive mental health literacy and mental well-being among adolescents who did and did not participate in the MEST program over the course of a school year	*Mental Health Promoting Knowledge (MHPK-10); Short Warwick-Edinburgh Mental Wellbeing Scale (SWEMWBS); Health Literacy for School-Aged Children (HLSAC)*.
Morgado *et al*., 2021	*Adolescents' Empowerment for Mental Health Literacy in School: A Pilot Study on ProLiSMental Psychoeducational Intervention*	Portugal	Cluster-randomized quasi-experimental pilot study	To evaluate the effectiveness of a psychoeducational intervention (ProLiSMental) in promoting mental health literacy among adolescents	*Mental Health Literacy Questionnaire* (MHLq) – Adolescent Version (A-MHLq), QuALiSMental – Portuguese Version
Yüksek *et al*., 2024	*Effectiveness of health literacy education on health literacy in early adolescence: A randomized controlled trial*	Turkey	Randomized controlled clinical trial with parallel groups	To evaluate the effectiveness of a health literacy education program in improving health literacy levels among early adolescents	*Health Literacy Scale for School-Age Children*
Costa *et al*., 2022	*A qualitative study exploring adolescents' perspective about Mental Health First Aid Training Programmes promoted*	Portugal	Qualitative, exploratory study	Exploring adolescents' perspectives on mental health first aid training programs led by nurses in secondary schools	Individual semi-structured interviews

Author, year of publication	Title	Country	Type of study	Purpose	Assessment / Instruments
	by nurses in upper secondary schools				
Öztürk *et al.*, 2023	*The effect of structured health promotion education given to adolescents on health literacy and health-promoting behaviors*	Turkey	Quasi-experimental study with a control group	To determine the effect of a structured health promotion education program on health literacy and the level of health-promoting behaviors among adolescents	*Health Literacy Scale for School-Aged Children*; *Adolescent Health Promotion Scale*
Wallace, 2016	*Managing Use of Over-the-Counter Medications in the School Setting*	United States of America	Professional practice report / applied descriptive study	To describe the implementation and management of over-the-counter medications in school settings, demonstrating how this practice can reduce disruptions to education, promote health literacy, and ensure safety and legal compliance.	Not applicable
Chen *et al.*, 2024	*Mobile Health-Based Health Literacy Weight ManagementIntervention Using Smart Devices for Adolescents:A Convergent Mixed-Methods Pilot Study*	Taiwan	Pilot study using a convergent mixed-methods approach (quantitative and qualitative)	To develop and evaluate the feasibility of a weight management intervention program based on mobile health (mHealth) literacy for adolescents, using smart devices during the COVID-19 pandemic	*Health Literacy Weight Management Scale; Weight Management Health Behavior Scale*
Henry, 2020	*Using play to educate children about asthma*	United Kingdom	A report on practice and innovation in community nursing, based on a quality improvement project and descriptive qualitative evaluation	To describe and evaluate an innovative game- and story-based health literacy approach (*BreathChamps*) to educate school-age children about asthma and improve treatment adherence and self-management of the condition	*Asthma Control Questionnaire* (ACQ); Assessment of children's and parents' knowledge through questions asked at public events and recording of correct/incorrect answers; Observation of peer engagement and knowledge sharing.
Khanal, 2023	*Improving adolescent health literacy through school-based health literacy intervention: a mixed-method study protocol*	Nepal	Quasi-experimental study using a mixed-methods approach	To identify the health literacy needs of adolescents in schools in Nepal. To design, implement, and evaluate a school-based health literacy education intervention to	*HLS-Child-Q15-Nepal* (adapted and validated version)

Author, year of publication	Title	Country	Type of study	Purpose	Assessment / Instruments
				improve health literacy and the intention to engage in health-promoting behaviors	
Salkım *et al*., 2024	*The Effect of Mental Health Literacy Web-Based Education Initiative on Students' Knowledge Level, Help Seeking, and Stigmatizing Attitudes: A Randomize Controlled Study Protocol*	Turkey	Experimental study (randomized controlled trial)	To evaluate the effectiveness of an online educational intervention on mental health literacy among adolescents, focusing on knowledge levels, help-seeking attitudes, and stigma	*Child and Adolescent Mental Health Literacy Scale* (CAMHLS); *Attitudes Towards Seeking Psychological Help Scale – Short Form* (SPHS-S); *Self-Stigma About Seeking/Getting Psychological Help Scale* (SSOSH)
Henry, 2019	*Learning through play: using evidence to improve child asthma care*	United Kingdom	Critical reflection based on professional practice	To explore how learning through play and storytelling can improve health literacy and adherence to asthma treatment in children, by engaging children, families, and communities	Not applicable
Sousa *et al*., 2024	*Adolescents and parents coping with a parent's cancer: developing a nursing intervention model*	Portugal	Multi-method study (including a *scoping review* and four qualitative studies)	To develop a nursing intervention model for adolescents and parents coping with a parent's cancer, grounded in nursing theories and the concept of health literacy	Not applicable
McDonald *et al*., 2015	*Evaluation of a Resource for Adolescents and Young Adults Diagnosed With Cancer*	Australia	Quasi-experimental quantitative study evaluating an educational resource	To assess the impact of the book "N*ow What...? A Young Person's Guide to Dealing With Cancer*" on satisfaction, changes in knowledge, *coping*, and its relationship with health literacy levels, as well as its impact on stress levels among adolescents and young adults diagnosed with cancer	*Functional, Communicative and Critical Health Literacy measure* – FCC-HL, tailored to the oncology context
Mizobata *et al*., 2025	*Effectiveness of a Perioperative Pediatric Nursing Intervention with a Brazilian Educational Comic Book: A Quasi-Experimental Study*	Brazil	Randomized controlled trial: an experimental study with a control group	To evaluate the effectiveness of a nursing education intervention, based on a comic book story, in reducing anxiety and promoting health literacy among children undergoing elective surgery and their parents	*State-Trait Anxiety Inventory for Children* (STAI-C); *State-Trait Anxiety Inventory* (STAI) for parents; Knowledge questionnaire on the surgical

Author, year of publication	Title	Country	Type of study	Purpose	Assessment / Instruments
					procedure, developed and validated by the authors
Hanbaly *et al*., 2022	*Effect of Nursing Intervention on Juvenile Diabetic Patients' Health Literacy*	Egypt	Quasi-experimental study	To evaluate the effect of a nursing intervention on the health literacy of adolescents with type 1 diabetes, with a focus on improving knowledge, self-management skills, and healthy behaviors	*Health Literacy Measure for Adolescents (HLMA)* *Diabetes Knowledge Test*
Nagel *et al*., 2008	*Using Plain Language Skills to Create an Educational Brochure About Sperm Banking for Adolescent and Young Adult Males With Cancer*	Canada	Project on the development and initial evaluation of educational materials	To develop and implement an educational brochure written in plain language about semen cryopreservation for male adolescents and young adults with cancer, to help them understand the topic, promote informed decision-making, and reduce anxiety about the process	Not applicable
Jones *et al*., 2020	*A Digital Intervention for Adolescent Depression (MoodHwb): Mixed Methods Feasibility Evaluation*	United Kingdom	Feasibility study using a mixed-methods approach (quantitative and qualitative)	To assess the feasibility, acceptability, and potential impact of the *MoodHwb* digital program for adolescents with depression or at risk	*Adolescent Depression Knowledge Questionnaire* (ADKQ); Interviews; Surveys on program usage
Güven *et al*., 2020	*Evaluation of the efficiency of the web-based epilepsy education program (WEEP) for youth with epilepsy and parents: A randomized controlled trial*	Turkey	Experimental study (randomized controlled clinical trial)	To develop, test, and evaluate the effectiveness, quality, content, and usability of an online education program on epilepsy (*WEEP*), designed for young people with epilepsy (ages 9–18) and their parents/caregivers, to improve knowledge, self-efficacy, attitudes, and e-health literacy (*e-Health Literacy*)	*Epilepsy Health Literacy Scale* (EHLS), validated for adolescents; *Epilepsy Knowledge Questionnaire* (EKQ); *e-Health Literacy Scale* (eHEALS)
Alexander, 2020	*Supporting food literacy among children and adolescents: Undergraduate students apply public health nursing principles in clinical practice*	United States of America	Descriptive Study	To describe how nursing students apply public health nursing principles in promoting food literacy among children and adolescents	Not applicable

Author, year of publication	Title	Country	Type of study	Purpose	Assessment / Instruments
Barbazi *et al*., 2025	*Exploring Health Educational Interventions for Children With Congenital Heart Disease: Scoping Review*	United States of America	Scoping Review	To map and analyze health education interventions targeting children with congenital heart disease, identifying types, target populations, delivery methods, and evaluated outcomes, as well as gaps and priorities for future research.	Interviews, questionnaires, knowledge scales, behavioral observation
Francis *et al*., 2022	*Lessons learned from implementing SNAP-Ed in a nursing/K-8 partnership school during the pandemic*	United States of America	Reflective and descriptive experience narrative	Describe the lessons learned from the virtual implementation of the *SNAP-Ed* program to promote nutrition literacy among preschool-aged children during the COVID-19 pandemic	Not applicable
Kühne *et al*., 2025	*Investigating Primary School Nurses' Activities That Are Effective in Health Promotion and Primary Prevention: A Systematic Review*	Germany	Systematic Review	To identify the health promotion and primary prevention activities carried out by school nurses in elementary schools, and to assess which of these are effective in improving students' health outcomes	Self-report questionnaires, pedometers, assessments by nurses and other professionals, school records, environmental data (e.g., vaccination rates, dietary intake)
Pinto *et al.*, 2022	*Effectiveness of the Portuguese version of Fume on health literacy among teens and tobacco*	Portugal	Quasi-experimental study with pre- and post-test assessments and a control group	To evaluate the effectiveness of the Portuguese version of the digital educational game "Fume" in improving adolescents' health literacy regarding tobacco, by promoting knowledge and preventive attitudes toward tobacco use	Not applicable
Bıdık et al., 2021	*School-based mental health programs for improving psychosocial well-being in children and adolescents: Systematic review*	Turkey	Systematic review	To evaluate the results of school-based randomized controlled trials conducted with the aim of promoting the psychosocial well-being of children and adolescents and to assess the role of nurses in this type of intervention	Questionnaires on mental health literacy (knowledge, beliefs, and attitudes); Stigma and acceptance scales; Assessment of intention or behavior regarding help-seeking
McLelland *et al.*, 2021	*Effective HPV vaccination with Māori male students:*	New Zealand	Quality Assessment / Practice Report	To evaluate a *Kaupapa Māori* community initiative aimed at increasing HPV vaccination coverage among *Māori* male	Group discussion with eight students; Semi-structured interviews with key

Author, year of publication	Title	Country	Type of study	Purpose	Assessment / Instruments
	Evaluation of a Kaupapa Māori primary health care initiative			students (ages 13–17) in rural areas by promoting health literacy and vaccination adherence	informants; Reflective observations
Nogueira *et al.*, 2023	*Promoting literacy and positive mental health: A quasi-experimental study with adolescents in a school setting*	Portugal	Quasi-experimental study with pre- and post-test assessments and no control group	To promote positive mental health literacy and develop personal and social skills among adolescents through a structured program in a school setting	Positive Mental Health Literacy Questionnaire (adapted version); Personal and Social Competence Scale
Coelho, 2019	*Adolescents, Sexuality, and Health Literacy: A Community Nursing Intervention in a School Setting (Internship Report)*	Portugal	Community intervention project in a school setting	To promote health literacy and healthy sexual practices among adolescents, strengthening their ability to make responsible decisions and prevent risky behaviors, particularly those related to sexually transmitted infections and HIV	Sexuality Knowledge Questionnaire
Park *et al.*, 2018	*Youth Substance Use Prevention Using Disciplinary Literacy Strategies*	United States of America	Quasi-experimental pilot study with pre- and post-tests, of a quantitative and evaluative nature	To develop and test the feasibility of a community-based program for the prevention of substance use among adolescents, based on disciplinary literacy strategies applied to health education	The *Health Rocks! Intervention Survey* (assesses knowledge, skills, and personal resources); the *Virginia Tobacco Settlement Foundation* (VTSF) *Survey* (assesses smoking cessation intentions, perceptions of the harmfulness of smoking, perceived benefits, and attitudes toward smoking)

COVID-19 - Coronavirus Disease of 2019; HIV - Human Immunodeficiency Virus; HPV - Human Papillomavirus; MEST - short version of the Norwegian word for coping

Table S2 - Key features of nursing intervention to promote health literacy in children and adolescents

Study	Participants (Population)			Intervention						
	Age group	Characteristics	Sample size	Name	Theme	Duration, frequency	Methodology / Procedures / Activities	Materials used	Professionals involved	Context
Dülger *et al.*, 2024	Teenagers Ages 10–13 (Average: 12.6 years)	6th and 7th grade students in a school setting, with no health conditions preventing their participation, and with internet access	114 adolescents (42 in the experimental group, 72 in the control group, after attrition during the study)	Not identified	Health Literacy, Nutrition, Physical Activity	Duration: 10 weeks (6 theoretical modules, followed by 2 practical training modules, with assessment at three points: pre-test, post-test (2 months), and follow-up (5 months))	Self-paced online education featuring multimedia content, hands-on activities, reinforcement of healthy behaviors, and communication via *WhatsApp*; 4 theoretical modules designed to provide information: "Adolescence," "Lifestyle," "Nutrition," and "Physical Activity and Exercise" + 2 active training modules to reinforce positive behaviors: "I Know What I Eat, I Eat Healthy" (nutrition) and "Move to Feel Happy" (physical activity and exercise)	Online educational platform, WhatsApp for communication; digital quizzes; theoretical materials and interactive online activities	Nurses	School, but *online* (due to the COVID-19 pandemic)
Son *et al.*, 2017	Teenagers (Average: 16.2 years)	Youth in juvenile detention, considered a high-risk group (risky sexual behaviors, limited prior access to sex education)	134 young people (118 males, 16 females)	Not identified	Sexual Health	3 days (interactive curriculum)	Interactive small-group sessions, guided discussion, practical exercises, quizzes, *role-playing*; At least one physician or nurse specialist present to provide support and clarification; Topics: reproductive anatomy, sexual transmitted diseases, contraception, communication skills, decision-making, safe relationships	Curriculum adapted from Family Life and Sexual Health and Teens for AIDS Prevention; questionnaires; printed teaching materials; presentations and visual aids.	Collaboration among nursing, medical, and social work students, under the supervision of healthcare professionals (nurses	Institutional environment of juvenile justice

Study	Participants (Population)			Intervention						
	Age group	Characteristics	Sample size	Name	Theme	Duration, frequency	Methodology / Procedures / Activities	Materials used	Professionals involved	Context
									and physicians)	
Derwig *et al.*, 2021	4 and 5 years old	Children (and their families) who regularly use child health services in Sweden	203 children (intervention group); 582 children (control group)	*Child-Centred Health Dialogue* (CCHD)	Nutrition	Included in routine pediatric checkups	Part 1 (Universal): a single structured dialogue during a recommended consultation at age 4, using eight interactive illustrations that address healthy eating habits and lifestyles; Part 2 (Targeted): for children identified as overweight or obese, conducted 1 to 3 weeks after the initial visit, focused on a family therapy approach (Standardized Obesity Family Therapy - SOFT)	8 educational illustrations (fruits and vegetables, portion sizes, sugary drinks, physical activity, sedentary behavior, sleep routines, dental hygiene, etc.), a BMI chart, an instruction manual, and a standardized dialogue guide.	General Section – Nurses only; Specialized Section – support from nutritionists and pediatricians	*Child Health Centres*
Yoos *et al.*, 1997	Ages 2–18	Children living in urban areas who belong to ethnic minorities (primarily African Americans) and have moderate to severe asthma (and their families)	Approximately 40 families were interviewed; 12 children and adolescents, along with 7 parents, participated in a *focus group*	Asthma management program for children from ethnic minority groups in urban settings	Management of Chronic Diseases (Asthma)	Not identified	Educational sessions integrated into regular clinic visits and community events; Development of culturally sensitive educational materials adapted to literacy levels (5th to 7th grade); Organization of educational activities in community settings (schools, clinics, summer camps for children with asthma), taking advantage of existing opportunities (e.g., annual flu vaccination campaigns)	Written educational materials; Specific, tailored educational brochures on symptoms, medication, asthma triggers, and environmental control; A comprehensive	Pediatric nurses, in collaboration with doctors and social workers	Hospital pediatric clinic; School; Community (asthma camps, etc.)

Study	Participants (Population)			Intervention						
	Age group	Characteristics	Sample size	Name	Theme	Duration, frequency	Methodology / Procedures / Activities	Materials used	Professionals involved	Context
								educational workbook; Standardized forms for documenting the health care and education provided during clinic visits		
Almquist *et al.*, 1998	From birth to age 10 (at one clinic, but at the second clinic from ages 0 to 5)	Children receiving regular pediatric care, including families with limited resources and potentially low literacy levels	Not identified	*Children's Reach Out And Read*	Emerging Literacy	Duration: ongoing, incorporated into all well-child visits; Frequency: at each well-child visit, the child receives a book	Healthcare professionals provide new books appropriate for the children's ages; Volunteers read to the children and demonstrate reading aloud techniques in waiting rooms; Parents are given guidance on how to use the books to support emotional and cognitive development and promote healthy habits; Encouragement to use public libraries	New books tailored to the age group and reflecting cultural diversity; Used and new books donated by participants and the community; Informational brochures and practical guidance on literacy and early reading; Special bookcases (*"Share a Book, Share a Dream"*)	Nurses; Doctors; Volunteers	Clinics
Atkinson *et*	6 months to 5 years	Preschool-aged children, especially	Not identified	*Children's Reach*	Emerging	Duration: during well-child visits	Distribution of age-appropriate books during appointments; Reading aloud and modeling	New, high-quality, and culturally	Pediatric nurses; Volunteer	Pediatric primary care clinics

Study	Participants (Population)			Intervention						
	Age group	Characteristics	Sample size	Name	Theme	Duration, frequency	Methodology / Procedures / Activities	Materials used	Professionals involved	Context
al., 2002		those from low-income families with low functional literacy		*Out and Read*	Literacy	from 6 months to 5 years of age; Frequency: at each well-child visit	reading behavior by healthcare professionals; Volunteers read aloud in waiting rooms, demonstrating reading techniques to parents; Conversations with parents about the importance of daily reading, encouraging library use, and monitoring language development; Bilingual and culturally sensitive materials; Training and certification of clinics by the*ROR National Center.*	appropriate books (available in 12 languages); Reading guides for parents and *"reading prescription pads"*; Posters, videos, and educational brochures; *ROR* implementation manuals and checklists for professionals.	readers; Pediatricians; Educators	
Stonbraker *et al.*, 2020	Ages 14 to 19	Teenage mothers (mostly first-time mothers), the majority of whom are unemployed, financially dependent on their partners, and many of whom are still in school	58 teenage mothers participated in the intervention; 44 completed all phases (including follow-up)	Empowering Teenage Mothers	Maternal and Child Health	Duration: 12 weeks; Frequency: daily messages (weekdays)	Organized groups on *WhatsApp*, moderated daily by professionals; Quizzes; Discussion forums; An in-person introductory session (handing in cell phones, rules, start of activities) and an in-person final session (evaluation, reflection); Main topics: infant growth and development, breastfeeding, nutrition in the first year, maternal health, family planning methods, debunking myths	Cell phones and data plans; An educational manual developed specifically for the group; Informative text messages and images on *WhatsApp*; Online quizzes and group forums; A structured moderation guide	Nurse in charge of the study; Collaboration with physicians and other professionals	Community – digital engagement (via *WhatsApp*)

Study	Participants (Population)			Intervention						
	Age group	Characteristics	Sample size	Name	Theme	Duration, frequency	Methodology / Procedures / Activities	Materials used	Professionals involved	Context
Thomas, 2003	10 and 18 years old	Girls at risk of violence or already involved in aggressive behavior; Victims of *bullying*, domestic violence, or with low school engagement	Not identified	Program as literacia emocional: *Second Step*, RIPP (*The Responding in Peaceful and Positive Ways*), *The Ophelia Project*, *''Teaching Kids to Cope''*	Emotional literacy	Not identified	Three complementary approaches: (1) Emotional literacy and school violence programs; (2) Parenting education; (3) Individualized psychosocial intervention	Workshops and group educational sessions (prevention program); role-playing, art, peer mediation and parental involvement, skits, videos, debates, games, group discussions, and more	School nurses; Mental health nurses; School psychologists; Teachers; Parents	School; Community
Bereznay *et al*., 2019	Ages 13–18	Adolescents (community), especially those at risk of obesity or poor eating habits	Not applicable	Various programs	Nutrition	Not applicable	Weekly hands-on cooking classes; Use of technology (apps such as *Food Record* or *FuelUp&Go!*); Participation in programs such as SNAP-Ed, *Team Nutrition*, and *Slow Food USA*®; Participatory, interactive, and sensory-based methodologies	Materials from the SNAP-Ed and USDA Team Nutrition programs; Cookbooks; Posters; Measuring kits and kitchen utensils; Apps and interactive digital	Pediatric nurses in collaboration with community and school organizations	School; Community (churches, youth clubs)

Study	Participants (Population)			Intervention						
	Age group	Characteristics	Sample size	Name	Theme	Duration, frequency	Methodology / Procedures / Activities	Materials used	Professionals involved	Context
								resources; Educational guides and brochures on food labeling.		
Kemble *et al*., 2024	5 to 24 years old	Children and adolescents diagnosed with asthma, ranging from mild to severe	Not applicable	Nursing strategies for asthma self-management	Management of Chronic Diseases (Asthma)	Not applicable	Individual and group educational sessions of varying frequency and duration (ranging from single sessions to programs lasting several weeks); Mobile apps for monitoring, medication reminders, and symptom tracking; Structured programs featuring interactive illustrations and frequent communication with nurses for personalized follow-up; (Among others)	Interactive mobile apps; Educational illustrations on asthma management; Personalized action plans; Tools for monitoring symptoms (e.g., *Peak Flow Meter*); Written and visual educational materials	Nurses (in some studies in collaboration with other healthcare professionals)	Community; School; Hospital; Clinic (according to the studies analyzed)
Driessnack, 2009	9 years	He has a baby brother who was recently diagnosed with cystic fibrosis	1 illustrative case study	Not applicable	Management of Chronic Diseases (Cystic Fibrosis)	Not applicable	Using the *Harry Potter* story to explain patterns of recessive inheritance in cystic fibrosis	The Harry Potter Series	Pediatric nurses	Pediatric Nursing Consultation

Study	Participants (Population)			Intervention						
	Age group	Characteristics	Sample size	Name	Theme	Duration, frequency	Methodology / Procedures / Activities	Materials used	Professionals involved	Context
Kuzma *et al*., 2024	Children and adolescents (ages 10 to 17)	It works with adolescents in school, clinical, and community settings, but does not recruit participants directly	Not applicable	Not applicable	Sexual Health	Not applicable	Proposes integrated interventions during regular clinical visits (e.g., annual adolescent health checkups), using confidential interviews, tools such as HEADSSS (Home, Education, Activities, Drugs, Sex, Suicide), sharing of digital resources, and participatory discussions tailored to the young person's age and developmental stage; Other topics: healthy relationships and sexual consent, safe sexting	Not applicable	Specialist nurses (in some cases in collaboration with other professionals and with parental involvement)	Primary health care
Millar *et al*., 2024	Ages 6–14	Children and adolescents with cystic fibrosis and their families	Not applicable	"*Cystic Fibrosis Study Buddies Programme*"	Management of Chronic Diseases (Cystic Fibrosis)	Development and implementation of materials over several months, integrated into the regular clinical follow-up program for cystic fibrosis	Three priority areas in education: the importance of collecting respiratory secretions, nebulizer hygiene, and adherence to pancreatic enzyme replacement therapy	Storybooks and coloring books; Animated videos—featuring personalized health education (on the three priority areas of cystic fibrosis)	Multidisciplinary team: nurses, doctors, nutritionists, physical therapists, and psychologists	Hospital (pediatric setting specializing in cystic fibrosis)
Akca & Ayaz-	Ages 14–15	9th-grade students; high school	63 (divided into three	Not identified	Nutrition and Physica	Duration: 6 sessions, one per	Motivational interviewing: Groups of 7–8 students – open-ended questioning techniques,	PowerPoint presentations; Audiovisual	Nurses	School

Study	Participants (Population)			Intervention						
	Age group	Characteristics	Sample size	Name	Theme	Duration, frequency	Methodology / Procedures / Activities	Materials used	Professionals involved	Context
Alkaya, 2024		students without chronic illnesses, without specific dietary restrictions, who did not engage in regular exercise, and with low or moderate levels of health literacy	groups of 21)		1 Activity	week, 40 minutes each, held in a classroom	reflective listening, support, goal setting, decision-making assessment, motivation for change, etc.; Health education: 4 theoretical sessions and 2 practical sessions – oral presentations, Q&A, discussion, reading, videos, individual assignments, and practical demonstrations; Control group: no specific intervention; attended only the standard school health curriculum	materials (videos); Interview guides and teaching materials prepared by the researcher; *Smartwatch* provided to participants after the study as an incentive		
Watson & Serrant-Green, 2012	Ages 12–13	Female students attending a public school	74 teenagers	Educational Session on HPV	Sexual Health (HPV)	A one-time educational session lasting approximately 60–90 minutes	Activities: oral presentation, interactive discussion with the students, presentation of facts about HPV, modes of transmission, prevention, and the importance of vaccination	PowerPoint presentation; Informational brochures on HPV and vaccination; Educational visual materials (charts, explanatory images)	School nurses	School
Kostenius *et al*., 2021	Children (age not identified)	Students in 4th, 7th, and 9th grades, teachers, and school nurses	37 students, 12 teachers, and 44 school nurses	"*Health Dialogues*"	Various	Mandatory individual sessions with a school nurse at three	Health Dialogues: information, counseling, and learning tailored to each student's individual needs; They provide an opportunity for students to reflect on their health and their	Not applicable	School nurses	School

Study	Participants (Population)			Intervention						
	Age group	Characteristics	Sample size	Name	Theme	Duration, frequency	Methodology / Procedures / Activities	Materials used	Professionals involved	Context
						different stages of schooling	choices; They are seen as a way to build health-promoting relationships with students from preschool through high school; Completing health questionnaires *online*			
Bjørnsen *et al*., 2018	Ages 15 to 21	High school students of both genders	357 students (109 MEST participants and 248 non-participants)	MEST	Mental Health	Four educational sessions, each lasting approximately 60 minutes, held over four weeks during school hours	Topics covered: emotions and emotion regulation, social relationships, coping strategies, self-image, seeking help, and positive mental health; Active and participatory methods: group discussions, practical exercises, reflection activities and sharing of personal experiences, educational games; Materials adapted to the age group and cultural context of adolescents	MEST Manual (with guidelines and content for each session); Worksheets and supporting materials; Audiovisual materials (educational videos, presentation slides); Resources for practical exercises and group activities	School nurses; School psychologists	School
Morgado *et al*., 2021	Ages 14–15	9th-grade students; 63.2% female and 36.8% male	38 participants (21 in the intervention group and 17 in the control group)	ProLiSMental Psychoeducational Intervention	Mental Health	Four or eight weekly sessions of 90 minutes or 45 minutes, respectively	Participatory and dialogue-based methods, using videos, discussions, and group activities; Topics covered: Recognizing – understanding mental health, emotions, and anxiety; Caring – promoting mental health and strategies for prevention and self-help; Seeking help – mental health first aid, seeking informal	Structured outline of the ProLiSMental program. - QuALiSMental Questionnaire; Audiovisual and educational materials	Nurses specializing in: mental and psychiatric health, child and pediatric health, community	School

Study	Participants (Population)			Intervention						
	Age group	Characteristics	Sample size	Name	Theme	Duration, frequency	Methodology / Procedures / Activities	Materials used	Professionals involved	Context
							and formal help; Action: practical application of the knowledge gained		health (with experience in school health settings)	
Yüksek *et al*., 2024	Ages 12–13	Students in the 6th and 7th grades; adolescents of both genders who attended a public school and had a low level of health literacy at the outset (excluding those with a medium or high level)	80 adolescents (40 in the intervention group and 40 in the control group)	Health Literacy Education Program	Various	Duration: 40-minute sessions; Frequency: 4 educational sessions, one per week	Oral presentation, Q&A, group discussion, whisper technique, six thinking hats, case study	Computers; Projector; Whiteboard; Pencils and paper; Colored paper; Booklet with educational content distributed after the session	Nurses	School
Costa *et al*., 2022	Ages 15 to 18	Portuguese high school students (10th to 12th grade)	12 teenagers	Mental Health First Aid	Mental Health	Not applicable	The study did not evaluate a specific intervention, but collected data for the design of future programs; A variety of strategies are recommended (lectures, participatory activities, experiential learning, role-playing, and discussions); Preference is given to in-person sessions and raising awareness within the school community	Not applicable	Nurses	School (secondary education)

Study	Participants (Population)			Intervention						
	Age group	Characteristics	Sample size	Name	Theme	Duration, frequency	Methodology / Procedures / Activities	Materials used	Professionals involved	Context
Öztürk *et al.*, 2023	Ages 11 to 13	Elementary school students (6th, 7th, and 8th grades) at a public school, most of whom do not have chronic illnesses; from families with mostly middle-income levels	171 students (85 in the intervention group, 86 in the control group)	Not identified	Various	6 weeks (one module per week, lasting 1 hour)	6 modules: Nutrition, Exercise, Life Satisfaction, Stress Management, Social Support, Health Responsibility; Various techniques were used, such as direct instruction, question-and-answer sessions, video demonstrations, and games	38-page educational booklets covering structured health education topics and *kits* for personal hygiene, along with instructions on how to use the materials	Nurses	(Public) school
Wallace, 2016	Not identified	Students at a public school with a residential program, including students who live in dormitories during the week	Not applicable	Not applicable	Over-the-counter medication	Duration: during the assessment and administration of medications; Frequency: variable, depending on the need for care	Education on the proper use of over-the-counter medications; Addressing safety and preventing misuse; Promoting non-pharmacological strategies and healthy habits; Developing communication skills and health literacy	Digital and print educational resources	School nurses, in collaboration with the school physician	School (public school with a residential program)
Chen *et al.*, 2024	Ages 13 to 18 (average: 16.3 years)	Adolescents who were either overweight or obese; some participants had diagnoses	27 teenagers	"*GO! GO! Smart Healthy*"	Nutrition	Duration: 12 weeks; Frequency: ongoing monitoring and *feedback*	Mobile health app (mHealth *app*): Used by participants to provide data on weight management, eating habits, and health literacy; Divided into four sections: questionnaires, assessment of ideal weight,	Mobile health app (mHealth *app*): Used by participants to provide data on weight management,	Multidisciplinary team: pediatric nurses, school nurses, pediatrician	Schools (operating remotely or in a hybrid format during the

Study	Participants (Population)			Intervention						
	Age group	Characteristics	Sample size	Name	Theme	Duration, frequency	Methodology / Procedures / Activities	Materials used	Professionals involved	Context
		such as asthma (3.7%), congenital heart defect (3.7%), hypertension (3.7%), and allergic rhinitis (22.2%)					health education, and interactive sections; Health education tailored to each participant, addressing aspects of weight management relevant to their individual needs	eating habits, and health literacy; Divided into four sections: questionnaires, assessment of ideal weight, health education, and interactive sections; Health education tailored to each participant, addressing aspects of weight management relevant to their individual needs	s, nutritionists, family physicians, data engineers	pandemic), with individual use at home
Henry, 2020	Ages 7 to 11	Elementary school children, including some with asthma, though most interventions are aimed at all children to promote peer support and	Reported examples: 8 families at an elementary school (first pilot intervention); about 20 families at a community party; 12	"*Breath Champs*"	Management of Chronic Diseases (Asthma)	It depends on the methodology used	Methods: storytelling, role-playing (*Captain Fearless and the Wheeze Monsters*;*The Big Bad Wolf with Asthma*), interactive games, inhaler decoration, quizzes, songs, building lung models; Active participation, family involvement, and encouraging children to share what they have learned; Inhaler decoration contests; Educational songs.	Games and fun activities; Lung models; Ball-blowing contest; Interaction with puppets	Nurses	School; Community (community groups—e.g., parties, public events)

Study	Participants (Population)			Intervention						
	Age group	Characteristics	Sample size	Name	Theme	Duration, frequency	Methodology / Procedures / Activities	Materials used	Professionals involved	Context
		the dissemination of knowledge; some interventions also included families and educators	*Brownies* (Girl Scouts aged 7–9); Formal evaluation: 11 *Brownies* completed							
Khanal, 2023	Ages 13 to 19	Students in urban public schools, teachers, and school nurses	384 students	Health Literacy Educational Intervention	Various	8 sessions (1–1.5 hours each) over the course of a school semester	Competency-based sessions on LS (HLS-Child-Q15), preventive health, health literacy, service utilization, social determinants of health, etc	Interactive methods: role-playing, stories, videos, games, discussions	School nurses, teachers, public health physicians, and public health specialists	Schools (public)
Salkım *et al*., 2024	Ages 12 to 18	Elementary and secondary school students	100 students (50 in the experimental group, 50 in the control group)	*Web-Based Mental Health Literacy Education Initiative*	Mental Health	Assessments at pre-test, post-test (4 weeks), and follow-up (3 months)	6 modules, covering topics such as: What is mental health literacy?; What are mental illnesses? How are they treated?; The Human Brain—How is the Brain Linked to Development and Mental Health?; What is stigma and its effects on mental illness?; Why is it important to seek help and support?; Why is Positive Mental Health Important?; Each module is in video/animation format; individual access during the intervention period	*YouTube*(short videos, animations, audio presentations)	School nurses	School (public schools, with classes conducted *online*)

Study	Participants (Population)			Intervention						
	Age group	Characteristics	Sample size	Name	Theme	Duration, frequency	Methodology / Procedures / Activities	Materials used	Professionals involved	Context
Henry, 2019	Ages 4 to 11	Children with asthma	Not applicable	"*Asthma parties*"	Management of Chronic Diseases (Asthma)	1-hour sessions, school and community events	Play-based activities: games, songs, puppets, and crafts centered around stories (e.g., an adapted version of *The Three Little Pigs*—the wolf has asthma and learns to use an inhaler); Therapeutic and educational storytelling: children solve problems, take on roles, and teach the characters how to take care of their health; Simple, reusable teaching materials: dolls, puppets, paper tubes, balloons, and handmade instruments (e.g., making toy stethoscopes); Community involvement: training teachers, scout leaders, librarians, and parents to replicate the activities	Puppets and props inspired by children's stories; Recyclable materials (tubes, balloons, paper, tape); Educational videos and supporting materials (created by the author and volunteers).	Lead nurse, in collaboration with other healthcare professionals and community members (e.g., teachers)	Schools; Community; Local events; Primary care facilities
Sousa *et al.*, 2024	Ages 13–18	Adolescents with a parent who has cancer	Not identified (data derived from multiple qualitative case studies)	A nursing intervention model for adolescents and parents coping with a parent's cancer	Chronic Disease Management (Cancer)	Not applicable	Program structure covering 4 topics in individual and group sessions: 1 – "The elephant in the room": open discussion about a parent's cancer (individual session); 2 – "Everything changes within us": understanding and expressing the experience of a parent's cancer (individual session); 3 – "Life goes on": strategies for coping with parental cancer (group session); 4 – "With the present, we plan for the future": long-	Theoretical guides and protocols based on the Nursing Ontology; Educational resources on communication, emotions, and parenting; Welcoming and non-traumatic clinical spaces	Nurses specializing in pediatric health and mental health	Cancer hospitals (outpatient visits, day treatment, inpatient care)

Study	Participants (Population)			Intervention						
	Age group	Characteristics	Sample size	Name	Theme	Duration, frequency	Methodology / Procedures / Activities	Materials used	Professionals involved	Context
							term coping strategies and future impact (group session)	for individual or family sessions		
McDonald *et al.*, 2015	Ages 12 to 24 (average: 18.4 years)	Adolescents and young adults diagnosed with cancer, across various types of cancer and stages of treatment, supported by a national youth association (*CanTeen*, Australia)	46 young people	Educational resource "*Now What...? A Young Person's Guide to Dealing With Cancer*"	Chronic Disease Management (Cancer)	Preliminary review (before receiving the book) and follow-up review 2 months after reading the book	Delivery of the book (age-appropriate print version: ages 12–15 or 16–24); Free access to the book for two months, with the option to consult the sections deemed relevant at any given time; Addresses clinical, emotional, social, and practical issues related to the cancer journey (diagnosis, treatments, side effects, *coping*, relationships, the future, support contacts)	Printed educational book "*Now What...? A Young Person's Guide to Dealing With Cancer*" (two versions for two age groups); Digital version /*e-book*of the book	Nurses; Psychologists; Other healthcare professionals	*CanTeen*Australia (an organization that supports young people with cancer).
Mizobata *et al.*, 2025	Ages 6 to 12	Children in good physical health or with a mild systemic illness, undergoing their first elective surgery with an expected duration of up to two hours, whose parents are literate and able to	66 children and their parents	*"The Hospital Gang"*—an educational comic book about the pediatric perioperative process	Surgery	A single procedure performed the day before surgery, lasting an average of 20–25 minutes	The intervention took place the day before or on the day of surgery, during the preoperative assessment by the nurse; Control group: received only verbal information; Intervention group: received verbal information supplemented by reading and receiving the comic book, which could be consulted later	An educational comic book validated for the pediatric perioperative setting	Nurses	Hospital

Study	Participants (Population)			Intervention						
	Age group	Characteristics	Sample size	Name	Theme	Duration, frequency	Methodology / Procedures / Activities	Materials used	Professionals involved	Context
		communicate verbally								
Hanbaly *et al*., 2022	Ages 10 to 18	Young people of both sexes diagnosed with type 1 diabetes mellitus and followed at a pediatric endocrinology clinic	40 adolescents (20 in the experimental group and 20 in the control group)	Nursing Intervention Program for Young People with Diabetes	Management of Chronic Disease (Diabetes)	Total duration: 6 weeks	Weekly in-person sessions covering topics such as the disease, nutrition, physical activity, blood glucose monitoring, prevention of complications, and treatment adherence; hands-on activities, demonstrations, Q&A sessions, and training in self-management skills	Printed educational materials (brochures, posters, and guides); Equipment for demonstrating and practicing blood glucose monitoring; Daily health behavior log sheets; Visual and educational resources tailored to the participants' age group	Nurses	Hospital
Nagel *et al*., 2008	Ages 13–24	Male adolescents diagnosed with cancer	Not identified	*Educational Brochure on Sperm Banking for AYA Cancer Patients* (Educational Brochure on Sperm	Management of Chronic Diseases (Cancer)	Brochure provided during an oncology consultation, accompanied by a verbal explanation from a healthcare professional	Methodology: simple language, short sentences, everyday examples, *design* that appeals to the reader, questions and answers, logical sections	Educational brochure – "*Sperm Banking: Information for Teens With Cancer*"	Multidisciplinary team: pediatric oncology nurses, reproductive medicine nurses, social worker, health	Pediatric Cancer Center

Study	Participants (Population) Age group	Characteristics	Sample size	Intervention Name	Theme	Duration, frequency	Methodology / Procedures / Activities	Materials used	Professionals involved	Context
				Cryopreservation)					education specialist	
Jones *et al*., 2020	Ages 13 to 23 (average: 16)	Adolescents with depression or at high risk (e.g., family history)	44 young people; 31 parents/care givers; 13 professionals	"*MoodHwb*" (*HwbHwyliau* in Welsh, which means "*hub*of humor" or "emotional impulse").	Mental Health	Free access; Available for 2 months; 30 minutes per session	A digital platform featuring content on depression, self-management strategies, support resources, and a section for families; co-developed with teenagers and professionals	Web platform and mobile app (bilingual: English and Welsh); Animations, illustrations, and personal stories	Psychiatrists; Psychologists; School nurses; School counselors; Researchers in digital health	Online - Child and Adolescent Mental Health Services, schools, and youth organizations
Güven *et al*., 2020	Ages 9 to 18	Young people with epilepsy who attend follow-up appointments at the hospital; Without severe intellectual disability; Able to use a computer or smartphone with internet access; Parents or primary	70 (35 in the intervention group and 35 in the control group) and their parents	*Web-based Epilepsy Education Program* (WEEP)	Management of Chronic Disease (Epilepsy)	12 weeks, with continuous access to the platform and weekly reminders via text message	It included four interactive modules (one per week), covering topics such as epilepsy, self-care, emergency management, treatment adherence, myths, and stigma; Each module featured educational videos, presentations, interactive quizzes, and discussion forums; Participants had free access to supplementary materials and could ask questions of professionals through the platform	Interactive online platform (WEEP); Educational videos; Digital presentations; Online quizzes and forums; Digital educational materials (brochures, infographics)	Nurses working in collaboration with neurologists, psychologists, and IT specialists.	Online (Hospital - Pediatric Neurology Department)

Study	Participants (Population)			Intervention						
	Age group	Characteristics	Sample size	Name	Theme	Duration, frequency	Methodology / Procedures / Activities	Materials used	Professionals involved	Context
		caregivers of the young participants								
Alexander, 2020	Ages 5 to 13	Children and adolescents	640 children and adolescents	Not applicable	Nutrition	For 4 years	Nutrition and cooking education, featuring various *workshops*: "*MyPlate*" – meal preparation and cooking, with an emphasis on hand hygiene, safe food handling, and cutting techniques; *Workshops* on grocery shopping, using the "*Cooking Matters at the Store*" program; Interprofessional education program "*Culinary Medicine*," focused on economical, plant-based culinary strategies; Outdoor activities such as gardening to increase fruit and vegetable consumption	Kitchen and gardening supplies; Educational games, posters, quizzes, and reflective journals	Nursing students under the supervision of nursing faculty	School
Barbazi *et al.*, 2025	Ages 3 to 18	Children diagnosed with congenital heart disease (various types and degrees of severity); some studies also included family members/caregivers and healthcare	Not applicable	There is no single name, since the review covers various programs and interventions, highlighting examples	Management of Chronic Disease (Congenital Heart Disease)	The duration and frequency vary depending on the intervention (ranging from single sessions to programs lasting several weeks)	It depends on the type of training method used. Activities include lectures, games, simulations of everyday situations, hands-on demonstrations, case studies, and the use of videos and interactive mobile apps	Age-appropriate educational brochures and manuals; Board games and digital games; Mobile apps on self-care; Educational videos; Interactive and educational visual materials	Nurses; Doctors; Psychologists (in some interventions); Teachers (in school programs); IT technicians (in digital interventions)	Variable

Study	Participants (Population)			Intervention						
	Age group	Characteristics	Sample size	Name	Theme	Duration, frequency	Methodology / Procedures / Activities	Materials used	Professionals involved	Context
		professionals involved in the educational process		such as "*HeartSmart",* *"School-based Educational Program for CHD*", among others				(e.g., puppets, posters)		
Francis *et al.*, 2022	Ages 4 to 6	Students (from low-income families) in public schools	54 children (24 preschoolers and 30 elementary school students)	*Supplemental Nutrition Assistance Education Program* (SNAP-Ed)	Nutrition	9-month duration, once a month	Instructional videos/read-alouds of the *Read For Health* books (information on healthy eating), recipe sheets, family engagement calendars (a 7-day calendar with activities and information sharing for other household members), and documents ("*Tasting at Home*") to encourage young children's participation in cooking	*Chromebooks*	Graduate nursing students mentored by SNAP-Ed educators; Collaboration with teachers	*Online*, via Google Classroom
Kühne *et al.*, 2025	Ages 6 to 11	Primary school-aged children from different socioeconomic backgrounds; Some studies have focused on	Not applicable	Not applicable	Various	The interventions ranged from single sessions lasting 40–50 minutes to weekly programs lasting 6 to	Variable: interactive and creative activities (e.g., role-playing, group work, games), peer training, health screenings, and the provision of infrastructure (e.g., adequate sinks, posters)	Slide presentations, posters, brochures, text messages, oral hygiene materials, educational posters, prizes (e.g.,	School nurses; in some cases, teachers, other school staff, community health professional	School

Study	Participants (Population)			Intervention						
	Age group	Characteristics	Sample size	Name	Theme	Duration, frequency	Methodology / Procedures / Activities	Materials used	Professionals involved	Context
		communities at increased risk for certain diseases				12 weeks, or follow-up lasting up to 7 months		certificates, pedometers, water bottles)	s, and families	
Pinto *et al.*, 2022	Ages 10 to 13	Middle and high school students	144 teenagers (87 in the control group; 57 in the experimental group)	*No Fume*	Tobacco Prevention	A 20-minute guided gaming session + a recommendation to play for 2 weeks	The teenagers played individually, following the instructions and interacting with the different stages of the game; a fun and interactive activity that addressed topics such as the composition of tobacco, health risks, prevention, and cessation	Digital game "*No Fume*"; Computers or devices with access to the game	Nurses; Researchers in the fields of nursing and public health	School
Bıdık et al., 2021	5 to 18 years (depending on the study)	Elementary and secondary school students from diverse cultural and socioeconomic backgrounds in various countries	Not applicable	Not applicable	Mental Health	From one-time sessions to programs lasting several weeks	Interactive methods: lectures, group discussions, role-playing, videos, testimonials from people with experience in mental health	Presentations, educational videos, online resources, brochures, and visual materials tailored to the students' age group	School nurses (some studies); Teachers; Psychologists	School
McLelland *et al.*, 2021	Ages 13 to 17	Male students, mostly from local*Māori*tribes, living in rural areas	37 students	Not applicable	Sexual Health (HPV)	1-year term	Information sessions at the school, led by a male *Māori* doctor, with support from male Māori teachers; Vaccinations administered at the school by *Hauora* nurses; Inclusion of Māori cultural elements	Educational materials on HPV	Community nurses, a Māori male doctor, Māori male teachers, and a school principal	School

Study	Participants (Population)			Intervention						
	Age group	Characteristics	Sample size	Name	Theme	Duration, frequency	Methodology / Procedures / Activities	Materials used	Professionals involved	Context
Nogueira *et al.*, 2023	Ages 12 to 18 (average age 14)	High school students of both genders attending a public school	72 teenagers	+Mental Health	Mental Health	8 weekly sessions, 90 minutes each	Interactive and participatory activities: group exercises, role-playing, debates, group projects; Content: self-awareness, emotional management, effective communication, conflict resolution, decision-making, self-esteem, and life planning	Worksheets, PowerPoint presentations, educational videos, materials for group activities	Nurses specializing in mental health and psychiatry	School
Coelho, 2019	Ages 13 to 15	Middle school students of both genders attending a public school	40 teenagers	Adolescence with Wisdom – Living a Healthy Sexuality	Sexual Health	4 classroom sessions, each lasting approximately 90 minutes	Activities: group exercises, interactive discussion, viewing and analysis of educational videos, Q&A session; Content: changes during adolescence, sexual transmitted diseases prevention, contraceptive methods, responsible decision-making	PowerPoint presentations, educational videos, informational brochures, and models and examples of contraceptive methods for demonstration purposes	Nurses specializing in community nursing	School
Park *et al.*, 2018	Ages 10 to 15 (Average: 11.16 years)	32 adolescents from diverse ethnic backgrounds and from families with low socioeconomic status	32 teenagers / preteens	*Health Rocks!*	Prevention of Substance Use (Tobacco, Alcohol, and Drugs)	10 weeks, 1 hour per week, during the summer.	Structured around the *learning cycle* with three phases: pre-active (activating prior knowledge and setting objectives), interactive (reading multiple texts on tobacco/drugs using reading guides and *think-aloud*), reflective (role-play, writing letters to parents, group discussions, media criticism, anti-tobacco posters)	Curriculum *Health Rocks!*, reading materials, videos, reading guides, comprehension questions; creation of posters and letters	Public health nurse; Health educator; Youth center staff	Youth Community Center

BMI - Body Mass Index; e.g. - *exempli gratia;* HEEADSSS - Home, Education/Employment, Activities, Drugs, Sexuality, Suicide/Depression, and Safety; HPV - Human papillomavirus; IT - Information Technology; MEST - short version of the Norwegian word for coping; USDA - United States Department of Agriculture;
